# Ti_3_C_2_-Mxene-dispersion and morphology controlled battery-type nickel cobalt sulphide based nanocomposites for the application as aqueous asymmetric supercapacitor with improved rate

**DOI:** 10.1186/s11671-025-04396-3

**Published:** 2025-11-24

**Authors:** Abhinaba Das, Arnab Samanta Roy Choudhury, Pallab Bhattacharya

**Affiliations:** 1 Functional Materials Group, Advanced Materials & Corrosion (AMC) Division, CSIR-National Metallurgical Laboratory (NML), Burmamines, East Singhbhum, Jamshedpur, Jharkhand 831007 India; 2 Academy of Scientific & Innovative Research (AcSIR), Ghaziabad, 201002 India

**Keywords:** Nickel cobalt sulphide, Ti_3_C_2_ MXene dispersion, Hydrothermal synthesis, Morphology, Supercapacitor

## Abstract

Nickel cobalt sulphide (NiCo_2_S_4_) is a promising battery-type electrode material due to its high theoretical capacitance and rich redox activity. However, its poor electrical conductivity and structural instability hinder practical application. Incorporation of two-dimensional (2D) Ti_3_C_2_ MXene can address these issues by improving conductivity and mechanical integrity. While previous studies have explored the synergistic effects of Ti_3_C_2_ and NiCo_2_S_4_, the influence of MXene exfoliation state and morphology control on electrochemical performance remains underexplored. Herein, we report a one-step hydrothermal synthesis of delaminated Ti_3_C_2_@NiCo_2_S_4_ (d-Ti_3_C_2_@NiCo_2_S_4_) composites with tunable morphology by varying hydrothermal time (4–48 h). Among them, the 24 h sample (d-Ti_3_C_2_@NiCo_2_S_4_-24) featuring a hexagonal layered platelet structure exhibits superior performance, delivering 161.94 mAh g^−1^ (1165 F g^−1^) at 1 A g^−1^, with ~ 81% rate capability at 5 A g^−1^ and 85% capacity retention over 20,000 cycles. It significantly outperforms both bare NiCo_2_S_4_-24 and the multilayer Ti_3_C_2_-based composite. The asymmetric device (d-Ti_3_C_2_@NiCo_2_S_4_-24//AC) delivers 19.88 Wh kg^−1^ at 399.82 W kg^−1^ with 86% retention after 9000 cycles, demonstrating excellent potential for practical energy storage applications.

**Supplementary Information:**

The online version contains supplementary material available at 10.1186/s11671-025-04396-3.

## Introduction

Energy storage devices play a critical role in enabling the transition to sustainable energy systems by efficiently capturing and delivering power generated from renewable sources such as solar, wind, and hydroelectric energy. The storage systems, such as Lithium-ion batteries [1], Sodium-ion batteries [2], ammonium ion battery [3], Zinc-ion batteries [4], Sodium metal batteries [5], Solid-state batteries [6] are designed to mitigate the intermittent nature of renewable energy generation and ensure a stable and reliable power supply with high energy density. However, most of them suffer from low power density, limited cycle life, and high safety concerns. Therefore, supercapacitors [7] play a major role in safe and clean energy storage solutions with high power density and long life. Supercapacitors are generally classified into several types based on their charge storage mechanisms and configurations, including electrical double-layer capacitors (EDLCs), pseudocapacitors, hybrid supercapacitors [8], solid-state supercapacitors [9, 10], zinc ion capacitors [11–13], sodium ion capacitors [14]. They face inherent limitations such as low energy density, high self-discharge rates, low cell voltage, and limited long-term energy retention. To overcome these challenges, the use of aqueous-based electrolytes offers enhanced safety, while expanding the operating potential window is key to improving energy output. Therefore, this study focuses on the development of aqueous electrolyte-based asymmetric supercapacitors to achieve improved overall energy storage performance.

Transition metal sulfides, particularly bimetallic systems like nickel cobalt sulfide (NiCo_2_S_4_), have garnered substantial attention as high-performance electrode materials for supercapacitors owing to their multiple oxidation states (Ni^2+^/Ni^3+^ and Co^2+^/Co^3+^), high theoretical capacitance, and excellent redox kinetics [15–17]. Among various configurations, NiCo_2_S_4_ exhibits superior electrochemical activity compared to its monometallic counterparts (e.g., NiS or CoS), making it a promising battery type candidate for next-generation energy storage devices [17, 18]. Despite these advantages, practical deployment of NiCo_2_S_4_ electrodes remains hindered by several critical challenges [19]. Firstly, the intrinsic low electrical conductivity of sulfides limits their charge transfer kinetics and rate performance. Secondly, repeated electrochemical cycling induces structural degradation due to volumetric expansion/contraction, ultimately compromising long-term cycling stability [20, 21]. To address these limitations, hybridization with 2D conductive materials such as graphene [17, 22], MoS_2_ [23], MXene and many more have been extensively explored.

In this context, two-dimensional titanium carbide MXenes (especially Ti_3_C_2_) offer unique opportunities for enhancing the performance of sulfide-based electrodes. The Ti_3_C_2_ MXenes serve not only as charge transport scaffolds but also as platforms for uniformly anchoring active nanostructures [24–27]. When integrated with NiCo_2_S_4_, MXenes can mitigate agglomeration, buffer volume changes, and facilitate efficient ion/electron transport across the electrode–electrolyte interface [28]. In the context, several studies have demonstrated the synergistic benefits of NiCo_2_S_4_/Ti_3_C_2_ MXene composite electrodes used for energy storage applications. However, most of these works follow multistep and complex preparation methods, which may limit product yield and scalability. For instance, Wei et al. [29] first grew a Ni,Co based metal organic framework (MOF), by using metallic salts in the presence of 2-methylimidazole and dimethylformamide, which was further vulcanized in the next step to finally obtain the desired NiCo_2_S_4_ attached to Ti_3_C_2_ MXene composite with accordion-shaped nanosheets for studying electrochemical storage and nonlinear optical properties. In another work, Y. Luo et al. [30] first fabricated Ni,Co based layered double hydroxide (LDH) by using Ni,Co-salts and urea in the presence of N-methyl-2-pyrrolidone (NMP) via reflux and repeated centrifugation process. Then, the prepared LDH has been transformed into NiCo_2_S_4_/d-Ti_3_C_2_ hybrids through sulphuration under a double-temperature zone quartz tube furnace. Furthermore, NiCo_2_S_4_ nanoflower attached to Ti_3_C_2_ MXene has been prepared by using ammonia as the external reducing agent in ethanol–water mixture heated on an oil bath before proceeding for hydrothermal reaction [31]. Self-assembled Ti_3_C_2_@NiCo_2_S_4_ microspheres have been prepared through a two-step hydrothermal method where the urea complex is firstly prepared, which further set for sulphuration [32]. Spinel NiCo_2_S_4_ electrodeposited Ti_3_C_2_T_x_ MXene nanostructures have also been synthesized on various conductive substrates such as Cu-foil, Ni-foam and vertically grown nanosheets where NiCo_2_S_4_ grown on Cu-foil exhibits specific capacitance of just 167.28 Fg^−1^ at 4 Ag^−1^ and its device with Ti_3_C_2_ MXene produces energy density of 14.86 Whkg^−1^ and cycle stability of 5000 cycles with only 79% capacitance retention [33]. Hence, a common drawback among existing reports is the reliance on complex multi-step synthesis protocols, sacrificial templates, use of toxic or organic solvents, and a lack of emphasis on scalable, green, and morphology-tunable methods. Therefore, while Ti_3_C_2_ MXene and NiCo_2_S_4_ can provide synergistic benefits for electrochemical storage, the impact of hydrothermal synthesis duration on composite morphology and its correlation with electrochemical performance may open further avenues, as it is largely unexplored till now.

Therefore, in this work, we report a green, one-step hydrothermal synthesis of delaminated Ti_3_C_2_ MXene-modified NiCo_2_S_4_ (d-Ti_3_C_2_@NiCo_2_S_4_) composites using metal nitrates and thiourea in water, without additional reducing agents. The influence of hydrothermal duration (4–48 h) on the morphology and electrochemical performance was systematically investigated, revealing that d-Ti_3_C_2_@NiCo_2_S_4_-24, with its hexagonal plate-like structure, delivered the highest specific capacity 161.94 mAhg^−1^ (1165 Fg^−1^) at 1 Ag^−1^, excellent rate capability (~ 81% retention at 5 Ag^−1^), and remarkable cycling stability with 85% capacitance retention after 20,000 charge–discharge cycles. The asymmetric supercapacitor device (d-Ti_3_C_2_@NiCo_2_S_4_-24//AC) delivered a wide operating window of 1.6 V and achieved an energy density of (19.88) Whkg^−1^ at a power density of (399.82) Wkg^−1^, along with excellent cycling stability (86% retention after 9000 cycles). This work demonstrates a sustainable synthesis strategy with tunable morphology, enabling high-performance MXene-sulfide-based asymmetric supercapacitors.

## Experimental procedures

### Synthesis of Ti_3_C_2_ MXene dispersion (d-Ti_3_C_2_) from Ti_3_C_2_ MXene

The synthesis of Ti_3_C_2_ was reported in our previous work [24]. For the synthesis of d-Ti_3_C_2_ (dispersion of MXene), 0.2 g of Ti_3_C_2_ (particle size  ≤  38 µm) was dispersed in 80 mL of distilled water and ultrasonicated for 2 h to ensure uniform exfoliation. The suspension was then centrifuged at 3500 rpm for 20 min, and the supernatant, containing approximately 50 mg of Ti_3_C_2_ having a concentration of 0.63 mg/mL, was carefully collected for subsequent applications. Based on sediment–supernatant separation, the approximate ratio of few-layer to multilayer Ti_3_C_2_ (ML-Ti_3_C_2_) was estimated as 1:3 (calculation has been discussed in Supporting Information). The colloidal stability of the dispersion, evaluated using the Tyndall effect (Fig. S1), indicated that d-Ti_3_C_2_ maintained a stable greenish-hued suspension with no visible sedimentation for up to 24 h, whereas ML-Ti_3_C_2_ (prepared using bulk Ti_3_C_2_ MXene at the same concentration as d-Ti_3_C_2_) lost stability within 15 min. Moreover, ML-Ti_3_C_2_ exhibits an average lateral size of ~ 4.46 µm, whereas d-Ti_3_C_2_ shows ~ 1.67 µm, with TEM images of the flakes provided in Fig. S1c, g.

### Synthesis of d-Ti_3_C_2_@NiCo_2_S_4_

A metal precursor solution was prepared in a 1:2:13 molar ratio of Ni(NO_3_)_2_·6H_2_O, Co(NO_3_)_2_·6H_2_O, and thiourea, respectively. Specifically, 0.202 g of Ni(NO_3_)_2_·6H_2_O and 0.404 g of Co(NO_3_)_2_·6H_2_O were each dissolved in 2 mL of distilled water, while 0.7 g of thiourea was dissolved in 5 mL of distilled water. These precursor solutions were then added dropwise and slowly to the Ti_3_C_2_ MXene dispersion under continuous magnetic stirring to ensure homogeneous mixing and controlled nucleation of the sulphide phases. The entire mixture was further sonicated for 30 min to promote strong interaction between the precursors and the Ti_3_C_2_ MXene substrate. The final suspension was transferred into a Teflon-lined stainless-steel autoclave and subjected to hydrothermal treatment at 200 °C. To optimize the hydrothermal duration, the synthesis was carried out for different time intervals: 4 h, 16 h, 24 h, and 48 h. The resulting samples were designated as d-Ti_3_C_2_@NiCo_2_S_4_-4, d-Ti_3_C_2_@NiCo_2_S_4_-16, d-Ti_3_C_2_@NiCo_2_S_4_-24, and d-Ti_3_C_2_@NiCo_2_S_4_-48, respectively. During the reaction, the evolution of H₂S gas was noted, indicative of thiourea decomposition, and the solution pH was found to be approximately 9, suggesting alkaline conditions favorable for metal sulphide formation. After naturally cooling to room temperature, the resulting products were collected, thoroughly washed several times with distilled water and ethanol to remove residual ions and unreacted species, and subsequently dried in a vacuum oven. The final powders were stored in a desiccator for further physicochemical and electrochemical characterization.

### Synthesis of ML-Ti_3_C_2_@NiCo_2_S_4_

The synthesis was carried out using the same procedure as for d-Ti_3_C_2_@NiCo_2_S_4_-24, except that bulk ML-Ti_3_C_2_ MXene was used in place of the delaminated d-Ti_3_C_2_.

## Results and discussion

**Fig. 1:**
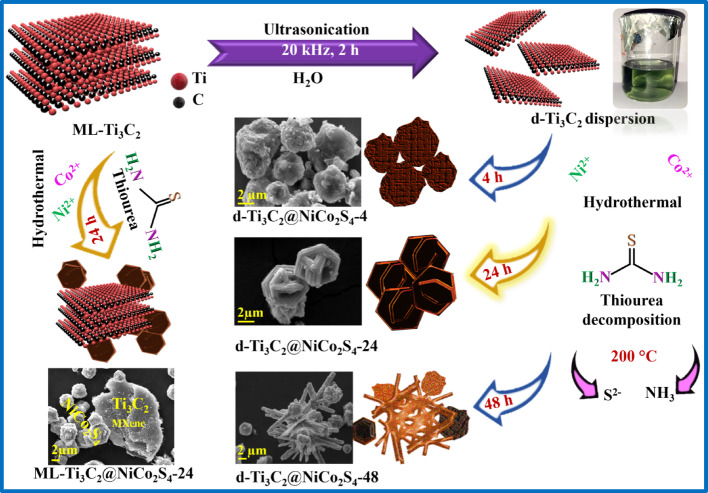
Schematic illustration of the formation of morphology-controlled d-Ti_3_C_2_@NiCo_2_S_4_ and ML- Ti_3_C_2_@NiCo_2_S_4_-24 composites

Figure 1 illustrates the synthesis route for d-Ti_3_C_2_@NiCo_2_S_4_ composites, specifically optimized for the 24 h hydrothermal treatment. Multilayered Ti_3_C_2_ MXene was first subjected to ultrasonic exfoliation (20 kHz, 2 h) in aqueous medium, followed by centrifugation at 3500 rpm for 15 min to obtain few-layered, delaminated Ti_3_C_2_ (d-Ti_3_C_2_). This exfoliated MXene was then utilized as a conductive scaffold in the hydrothermal synthesis of d-Ti_3_C_2_@NiCo_2_S_4_ composites using aqueous metal precursors and thiourea, without external reducing agents. To systematically investigate the influence of reaction time on the morphology and structure of the composite, hydrothermal treatments were conducted at 200 °C for 4 h, 16 h, 24 h, and 48 h. The morphological evolution at different durations was tracked using SEM analysis. After 4 h of hydrothermal treatment (d-Ti_3_C_2_@NiCo_2_S_4_-4), the product primarily exhibited spherical agglomerates (Fig. 1), indicating incomplete nucleation and growth. At 16 h (Fig. S2), the morphology began transitioning toward anisotropic structures, as partially developed hexagonal plates emerged. These features suggest the onset of directional crystal growth, likely driven by anisotropic energy minimization mechanisms [34, 35]. By 24 h, well-defined hexagonal plate-like structures with layered morphology were achieved (Fig. 1), which are highly beneficial for electrochemical applications due to their increased surface area and more accessible redox-active sites. However, extending the reaction time to 48 h (d-Ti_3_C_2_@NiCo_2_S_4_-48) led to a notable morphological transformation into mixed irregular rod-like along with deformed hexagonal structures (Fig. 1), attributed to Ostwald ripening and preferential growth along specific crystallographic directions during prolonged thermal exposure [36, 37]. This indicates that beyond a critical time threshold, structural destabilization and recrystallization processes dominate, adversely affecting the optimized morphology. These distinct growth mechanisms lead to variations in morphology without necessarily altering the phase (as confirmed later in the XRD section) [38]. To evaluate the role of MXene architecture in composite formation, a control sample was prepared using ML-Ti_3_C_2_ instead of d-Ti_3_C_2_, under identical hydrothermal conditions. The resulting ML-Ti_3_C_2_@NiCo_2_S_4_-24 composite exhibited poor morphological integration between the NiCo_2_S_4_ nanostructures and MXene layers (Fig. 1), suggesting limited interfacial contact and deficient ion/electron pathways. This inferior structural coherence ultimately leads to reduced electrochemical performance compared to its delaminated counterpart (d-Ti_3_C_2_@NiCo_2_S_4_), thus underscoring the importance of MXene exfoliation in achieving optimal composite morphology, charge transport, and overall capacitive performance (discussed in detail in later section).

**Fig. 2:**
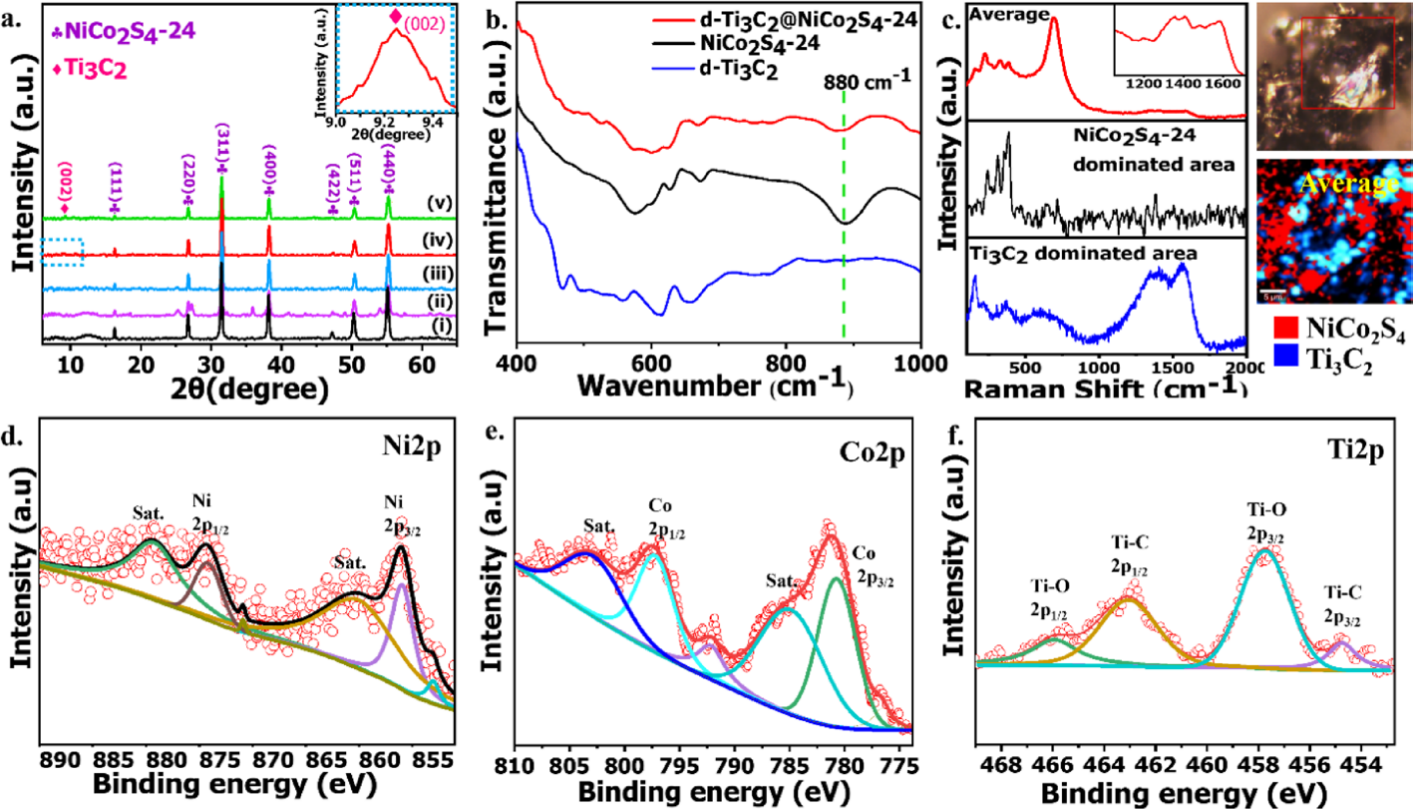
**a** XRD of (i) NiCo_2_S_4_-24, (ii) d-Ti_3_C_2_@NiCo_2_S_4_-4, (iii) d-Ti_3_C_2_@NiCo_2_S_4_-16, (iv) d-Ti_3_C_2_@NiCo_2_S_4_-24 (inset shows the magnified 002 peak of Ti_3_C_2_ MXene), (v) d-Ti_3_C_2_@NiCo_2_S_4_-48; **b** FTIR of d-Ti_3_C_2_@NiCo_2_S_4_-24, NiCo_2_S_4_-24, d-Ti_3_C_2_; **c** Raman spectra & mapping of d-Ti_3_C_2_@NiCo_2_S_4_-24; **d** XPS of Ni2p; **e** XPS of Co2p; **f** XPS of Ti2p

X-ray diffraction (XRD) analysis was carried out to examine the crystalline phases of the developed materials, as presented in Fig. 2a and Fig. S1d, h. The NiCo_2_S_4_-24 sample displays distinct peaks at 16.28°, 26.74°, 31.5°, 38.2°, 47.4°, 50.43°, and 55.2°, corresponding to the (111), (220), (311), (400), (422), (511), and (440) planes of spinel NiCo_2_S_4_ (JCPDS No. 20–0782) [28, 39]. XRD of ML-Ti_3_C_2_ and d-Ti_3_C_2_ (Fig. S1d, h) shows a broadened (002) peak with increased FWHM (from 0.46 to 0.60), a shift of ~ 0.16° toward lower 2θ, and an increase in d-spacing from 0.97 nm to 0.99 nm, indicating successful exfoliation and interlayer expansion in case of d-Ti_3_C_2_ as compared to ML-Ti_3_C_2_. The resulting d-Ti_3_C_2_@NiCo_2_S_4_ composites exhibit diffraction peaks located at nearly the same 2θ values as those of NiCo_2_S_4_-24, along with a broad (002) peak at ~ 9.25°, as highlighted in the inset (for d-Ti_3_C_2_@NiCo_2_S_4_-24), confirming the presence of Ti_3_C_2_ MXene which closely matches the diffraction pattern of the bare Ti_3_C_2_ MXene and is in good agreement with previously reported MXene structures [24, 40]. However, at shorter hydrothermal reaction times of 4 h (d-Ti_3_C_2_@NiCo_2_S_4_-4) and 16 h (d-Ti_3_C_2_@NiCo_2_S_4_-16), the NiCo_2_S_4_ peaks appear weak and are accompanied by additional small, unidentified peaks, indicating incomplete phase formation and the possible presence of intermediate or impurity phases. With increased reaction time, the diffraction peaks of NiCo_2_S_4_ become more defined, and at 24 h (d-Ti_3_C_2_@NiCo_2_S_4_-24) a clear and simultaneous presence of both NiCo_2_S_4_ and Ti_3_C_2_ MXene peaks is observed, reflecting successful composite formation with good crystallinity and structural integrity. However, further increasing the reaction time to 48 h (d-Ti_3_C_2_@NiCo_2_S_4_-48) results in the gradual suppression of the Ti_3_C_2_ (002) peak intensity, which is attributed to overgrowth or agglomeration of NiCo_2_S_4_ nanostructures on the MXene surface, possibly masking or disrupting the layered MXene structure. Importantly, the Ti_3_C_2_ (002) peak appears in all d-Ti_3_C_2_@NiCo_2_S_4_ composites and is absent in bare NiCo_2_S_4_, as shown in Fig. S3, confirming the incorporation of Ti_3_C_2_ in the composites, with a slight shift toward higher 2θ likely arising from partial restacking of the Ti_3_C_2_ MXene layers during composite formation. Based on these observations, the d-Ti_3_C_2_@NiCo_2_S_4_-24 composite demonstrates the most optimal phase composition and well-defined integration of both components, making it the most suitable for electrochemical energy storage.

The FTIR analysis has been carried out for the developed d-Ti_3_C_2_, NiCo_2_S_4_-24 and their composite d-Ti_3_C_2_@NiCo_2_S_4_-24 and presented in Fig. 2b. At the lower wavenumber (i.e. 400–1000 cm^−1^), all three FTIR spectra shows several peaks due to the presence of multiple transition metal (Ni, Co, and Ti) based vibrations. For example, in d-Ti_3_C_2_, the peak at 404–468 cm^−1^ mostly corresponds to Ti-C stretching E_u_ vibration [41], peaks in between 558 and 656 cm^−1^ correspond to Ti–O bending A_2u_ vibrations and the peak around 752 cm^−1^ appears due to Ti-F bending A_2u_ vibration [42]. On the other hand, the FTIR peaks for NiCo_2_S_4_-24 appeared at around 570 cm^−1^, 627 cm^−1^, 672 cm^−1^, and 880 cm^−1^ (symmetrical stretch) and 1092 cm^−1^ (asymmetrical stretch) are mainly caused by Ni–S or Co–S vibrations [25, 43]. Interestingly, the peak around 880 cm^−1^ is present in both NiCo_2_S_4_-24 and d-Ti_3_C_2_@NiCo_2_S_4_-24 composite but absent in d-Ti_3_C_2_, which indicates that the peak specifically appears from the Ni-S/Co-S vibrations [44].

Raman spectroscopy was further performed to analyze the local bonding and vibrational modes, aiding in understanding the heterostructure and crystallinity of the d-Ti_3_C_2_@NiCo_2_S_4_-24 composite is presented in Fig. 2c. Mapping over a relatively large area (80 × 80 micron) revealed that the d-Ti_3_C_2_@NiCo_2_S_4_-24 composite consists of regions dominated either by NiCo_2_S_4_ or by Ti_3_C_2_ MXene, with both phases coexisting throughout the material as evidenced by the average spectra. The Raman mapping illustrates the spatial distribution of NiCo_2_S_4_ and Ti_3_C_2_ MXene, with NiCo_2_S_4_-dominant regions highlighted in red exhibiting characteristic peaks arising mainly from vibrational modes associated with the S–Ni tetra and S–Co tetra bonds within the spinel structure. Notable peaks appear around 165, 242, 358, and 384 cm^−1^ as well as 461, 574, and 1017 cm^−1^, where the stretching of sulphur atoms towards the tetrahedral Ni sites and the bending of S–Ni_tetra_–S bonds give rise to the A_1__g_ (384 cm^−1^) and E_g_ (242 cm^−1^) modes respectively, while the T_2g_ modes (165, 314, and 358 cm^−1^) correspond to asymmetric bending of these bonds. In contrast, Ti_3_C_2_ MXene-dominant regions (shown in blue) exhibit peaks at 396 cm^−1^ and 607 cm^−1^ attributable to Ti–C bonds, with additional features at 156 cm^−1^ representing Ti–O stretching vibrations and peaks at 212 and 613 cm^−1^ corresponding to non-stoichiometric δ-TiC_x_ phases [45]. The D and G bands characteristic of MXene appear at 1387 cm^−1^ and 1568 cm^−1^ respectively [46]. The average Raman spectrum collected over the scanned area of the d-Ti_3_C_2_@NiCo_2_S_4_-24 composite combines the signatures of both NiCo_2_S_4_ and Ti_3_C_2_ MXene, including broad D and G bands centered at 1364 cm^−1^ and 1578 cm^−1^ (as shown in the inset), indicating that the intimate coexistence of these phases in d-Ti_3_C_2_@NiCo_2_S_4_-24 electrode may contribute synergistically to enhancing the overall energy storage performance.

XPS analysis has been carried out to further understand the chemical interaction in the developed d-Ti_3_C_2_@NiCo_2_S_4_-24 composite. The surface chemical composition of d-Ti_3_C_2_@NiCo_2_S_4_-24 composite was further examined through X-ray photoelectron spectroscopy (XPS) analysis. The full-range XPS spectrum (Fig. S4a) confirms the presence of Ni, Co, S, Ti, and C elements, indicative of the successful synthesis of the composite. Quantitative XPS survey analysis further shows that the d-Ti₃C₂@NiCo₂S₄-24 composite comprises ~ 12% Ni, ~ 22% Co, ~ 31% S, and ~ 8% Ti. Furthermore, A strong O 1s signal was also detected, which can be attributed to the inevitable surface oxidation that occurred during exposure of the samples to ambient air during the preparation process. This surface oxidation is a common phenomenon in transition metal sulphides and Ti_3_C_2_-based materials. The high-resolution Ni 2p spectrum (Fig. 2d) displays the characteristic peaks of both Ni^2+^ and Ni^3+^ oxidation states, along with two prominent satellite peaks (labeled “Sat”), reflecting the mixed-valence state of nickel in the NiCo_2_S_4_ phase [47]. This mixed-valence configuration is beneficial for redox activity, which can enhance electrochemical performance. The Co 2p spectrum (Fig. 2e) reveals two spin–orbit doublets corresponding to Co 2p_3/2_ and Co 2p_1/2_, located at 780.7 eV and 797.2 eV, respectively, along with two distinct satellite peaks. The presence of both Co^2+^ and Co^3+^ further suggests the coexistence of multiple oxidation states, which is consistent with the typical electronic structure of NiCo_2_S_4_ [48–50]. In the Ti 2p spectrum (Fig. 2f), four deconvoluted peaks are identified at binding energies of 465.8, 463.0, 457.7, and 454.6 eV. These peaks are assigned to Ti–O 2p_1/2_, Ti–C 2p_1/2_, Ti–O 2p_3/2_, and Ti–C 2p_3/2_, respectively, confirming the coexistence of both titanium carbide and oxidized titanium species on the surface [51]. The partial oxidation of Ti_3_C_2_ is typically due to its high surface reactivity in air, yet its retention of Ti–C bonding also demonstrates the structural integrity of the MXene component. The high-resolution C 1s spectrum (Fig. S4b) shows multiple peaks at 284.1 eV, 285.3 eV, 287.9 eV, and 289.3 eV, corresponding to C=C, C–C, C=O, and O–C=O bonds, respectively [52–54]. These signals reflect both the intrinsic graphitic carbon and surface functional groups introduced during synthesis or oxidation. Lastly, the S 2p spectrum (Fig. S4c) reveals two main peaks at 161.3 and 162.5 eV, corresponding to S 2p_3/2_ and S 2p_1/2_, respectively, confirming the presence of metal–sulphide bonding. Additionally, a broader peak near 168.4 eV is observed, which is attributed to oxidized sulphur species (SO_x_), indicating surface sulphur oxidation—likely due to atmospheric exposure [55, 56]. This observation aligns well with literature reports on the surface instability of sulphide phases in air. Collectively, these XPS results validate the successful synthesis and surface chemical composition of the d-Ti_3_C_2_@NiCo_2_S_4_-24 composite. The presence of multiple oxidation states in Ni and Co, along with the mixed chemical environment of Ti, S, and C, underscores the potential of this composite for applications in energy storage or electrocatalysis due to enhanced redox activity and surface reactivity.

**Fig. 3:**
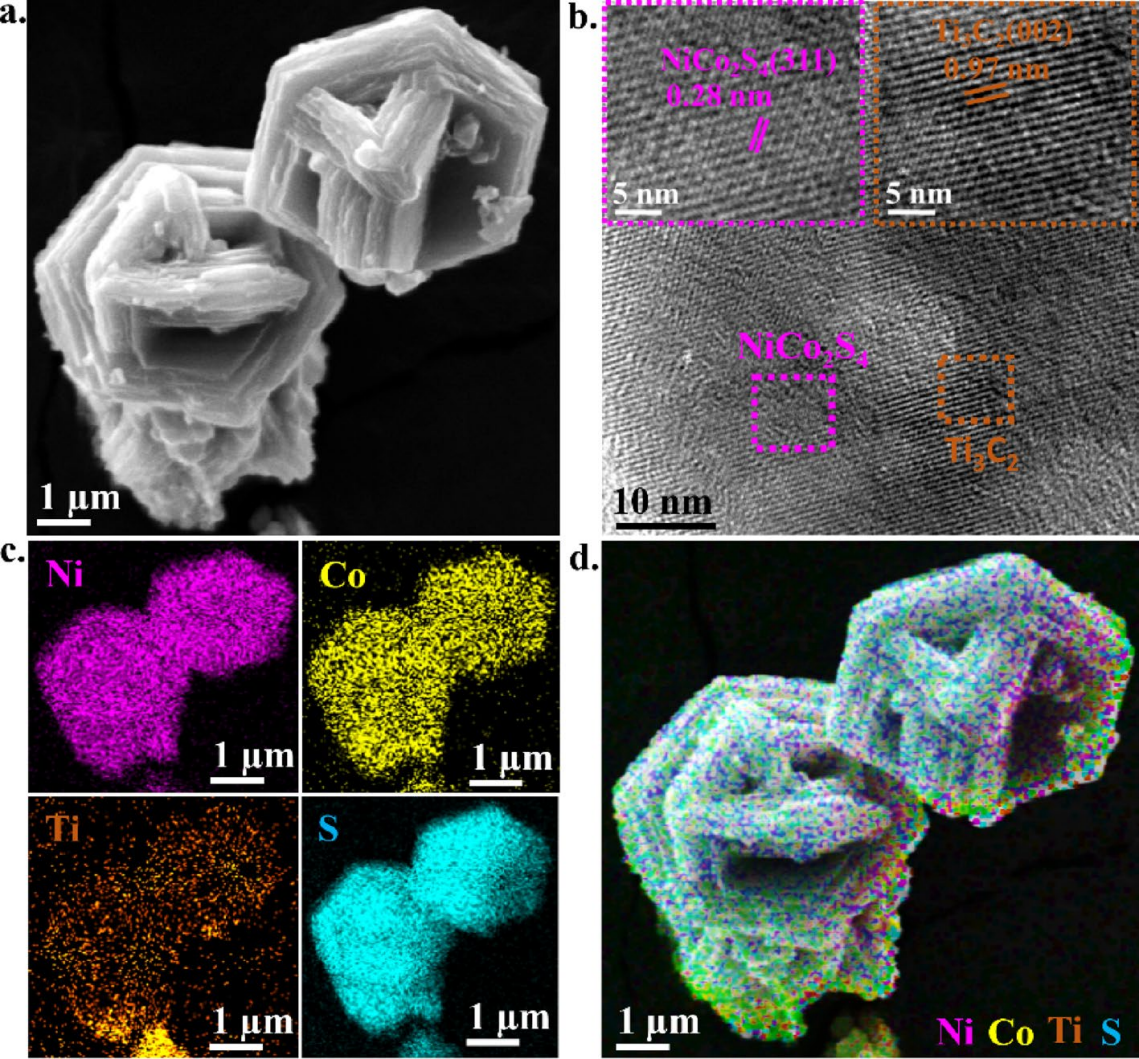
**a** SEM image of d-Ti_3_C_2_@NiCo_2_S_4_-24; **b** TEM image of d-Ti_3_C_2_@NiCo_2_S_4_-24; **c** and **d** EDS mapping of d-Ti_3_C_2_@NiCo_2_S_4_-24

The surface morphology of the synthesized d-Ti_3_C_2_@NiCo_2_S_4_-24 composite was further examined using SEM and HR-TEM techniques, as shown in Fig. 3. The SEM image (Fig. 3a) of d-Ti_3_C_2_@NiCo_2_S_4_-24 composite displays a hexagonal plate-like morphology with a visibly layered architecture, indicative of the presence of exfoliated Ti_3_C_2_ MXene coupled with NiCo_2_S_4_ nanostructures [57]. To gain deeper structural insight, HR-TEM analysis was performed (Fig. 3b), which further confirms the coexistence of both Ti_3_C_2_ MXene and NiCo_2_S_4_ phases. The inset shows two magnified regions distinctly marked in pink and orange, corresponding to NiCo_2_S_4_ and Ti_3_C_2_ MXene, respectively. The NiCo_2_S_4_ region exhibits well-defined lattice fringes with a d-spacing of 0.28 nm, corresponding to the (311) plane, while the Ti_3_C_2_ region shows a lattice spacing of 0.97 nm, characteristic of the (002) plane of Ti_3_C_2_ MXene. The SAED patterns (Fig. S5) of d-Ti_3_C_2_@NiCo_2_S_4_ composites prepared at different hydrothermal durations exhibit multiple concentric rings with bright spots, confirming their polycrystalline nature, primarily due to the overlapping contributions from Ti_3_C_2_ MXene and NiCo_2_S_4_ phases. These results correlate well with the XRD patterns, which consistently display the (311) plane as the most intense peak, suggesting preferential crystal growth along this facet. The intensity ratio of the (311) to (440) reflections i.e. I_(311)_/I_(440)_ increases from 1.42 (4 h) to 1.66 (16 h), peaking at 1.87 (24 h), and then slightly decreases to 1.74 (48 h). This evolution implies a dominant orientation along the (311) plane up to 24 h, promoting the formation of hexagonal platelet-like morphologies. At 48 h, increased contributions from the (440) plane lead to mixed morphologies, such as irregular rods and distorted hexagons. This observation aligns with previous studies [58, 59], which suggest that the (311) and (440) planes and their interplay can drive morphological diversity in NiCo₂S₄ systems. To further support the structural observations, elemental analysis was carried out through EDS mapping (Fig. 3c) on the same region imaged in the SEM Fig. 3a. The EDS maps confirm the homogeneous presence of Ni, Co, Ti, and S, which are the principal constituents of NiCo_2_S_4_ and Ti_3_C_2_. Moreover, the overall elemental distribution shown in Fig. 3d demonstrates a uniform dispersion of these elements across the d-Ti_3_C_2_@NiCo_2_S_4_-24 composite. Such compositional uniformity not only supports the successful integration of the two phases but also ensures consistent redox activity and efficient charge transport throughout the electrode, which are essential for high-performance electrochemical energy storage.

The nitrogen adsorption–desorption isotherms of NiCo_2_S_4_-24, d-Ti_3_C_2_@NiCo_2_S_4_-4, d-Ti_3_C_2_@NiCo_2_S_4_-16, d-Ti_3_C_2_@NiCo_2_S_4_-24, d-Ti_3_C_2_@NiCo_2_S_4_-48, and ML-Ti_3_C_2_@NiCo_2_S_4_-24 (Fig. S6a) exhibit typical type IV curves with H3 hysteresis loops, confirming the presence of mesoporous structures with corresponding BET surface areas of 0.5, 2, 6.5, 16, 3.7, and 2.5 m^2^ g⁻^1^, respectively. The d-Ti_3_C_2_@NiCo_2_S_4_-24 exhibits the highest BET surface area, possibly due to its hexagonal layered plate-like morphology. Moreover, the BJH pore size distribution profiles (Fig. S6b) reveal that all samples contain a combination of mesopores and macropores, with corresponding BJH pore volumes of 0.002, 0.003, 0.016, 0.028, 0.012, and 0.015 cm^3^ g^−1^, respectively, with d-Ti_3_C_2_@NiCo_2_S_4_-24 showing the highest surface area and pore volume.

The electrochemical behavior of the synthesized electrodes was evaluated using cyclic voltammetry (CV) in a three-electrode configuration with 6 M KOH as the electrolyte in the potential range of 0–0.5 V. The CV curve of NiCo_2_S_4_-24 (Fig. S7a), recorded at a scan rate of 2 mVs^−1^, exhibits a pair of broad and symmetric redox peaks, which can be attributed to the reversible faradaic redox transitions of nickel and cobalt species in an alkaline medium. The underlying redox mechanisms involve the following reactions [29, 48, 60]:

1 $$ {\text{NiCo}}_{{\text{2}}} {\text{S}}_{{\text{4}}} {\text{ + 2OH}}^{ - } {\text{ + 2H}}_{{\text{2}}} {\text{O}} \leftrightarrow {\text{NiSOH + 2CoSOH + 2e}}^{ - } $$

2 $$ {\text{CoSOH + OH}}^{ - } \leftrightarrow {\text{CoSO + H}}_{{\text{2}}} {\text{O + e}}^{ - } $$

3 $$ {\text{NiSOH + OH}}^{ - } \leftrightarrow {\text{NiSO + H}}_{{\text{2}}} {\text{O + e}}^{ - } $$

**Fig. 4:**
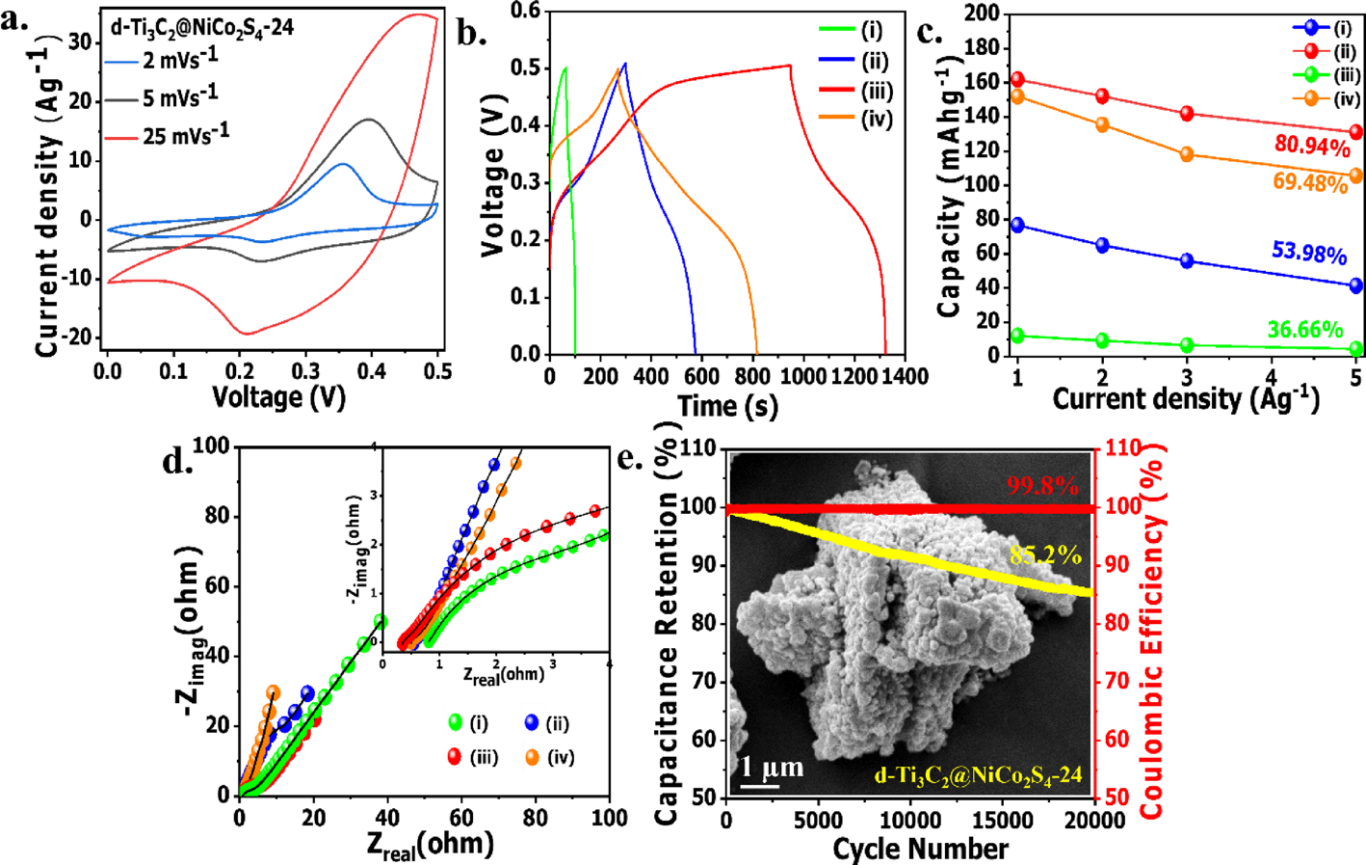
**a** CV of d-Ti_3_C_2_@NiCo_2_S_4_-24 in various scan rates; **b** GCD of (i) d-Ti_3_C_2_@NiCo_2_S_4_-4, (ii) d-Ti_3_C_2_@NiCo_2_S_4_-16, (iii) d-Ti_3_C_2_@NiCo_2_S_4_-24, (iv) d-Ti_3_C_2_@NiCo_2_S_4_-48; **c** variation of specific capacity with current densities of (i) d-Ti_3_C_2_@NiCo_2_S_4_-4, (ii) d-Ti_3_C_2_@NiCo_2_S_4_-16, (iii) d-Ti_3_C_2_@NiCo_2_S_4_-24, (iv) d-Ti_3_C_2_@NiCo_2_S_4_-48; **d** Nyquist plots of (i) d-Ti_3_C_2_@NiCo_2_S_4_-4, (ii) d-Ti_3_C_2_@NiCo_2_S_4_-16, (iii) d-Ti_3_C_2_@NiCo_2_S_4_-24, (iv) d-Ti_3_C_2_@NiCo_2_S_4_-48; **e** Cycle stability with coulombic efficiency of d-Ti_3_C_2_@NiCo_2_S_4_-24 and SEM image (inset) of d-Ti_3_C_2_@NiCo_2_S_4_-24 electrode after 20,000 cycles

These reactions contribute to a battery-type behavior governed by multi-step charge transfer and surface-controlled redox kinetics. In contrast, the CV curves of the Ti_3_C_2_ MXene-supported composites i.e., d-Ti_3_C_2_@NiCo_2_S_4_-4, d-Ti_3_C_2_@NiCo_2_S_4_-16, d-Ti_3_C_2_@NiCo_2_S_4_-48 and d-Ti_3_C_2_@NiCo_2_S_4_-24 (Fig. S7b and Fig. 4a) exhibit similar redox features, confirming that the charge storage mechanism remains fundamentally the same across all MXene-based samples. However, a notable difference lies in the integral area enclosed by the CV curves, with d-Ti_3_C_2_@NiCo_2_S_4_-24 displaying the largest area and highest current density at 25 mVs^−1^, highlighting the importance of hydrothermal time optimization in achieving superior electrochemical performance. The enhanced behavior of d-Ti_3_C_2_@NiCo_2_S_4_-24 is attributed to its hexagonal plate-like morphology with well-defined layered architecture, which facilitates efficient ion diffusion, rapid electron transport, and provides abundant electroactive sites for improved electrochemical performance. On the other hand, the CV curve of ML-Ti_3_C_2_@NiCo_2_S_4_-24 at 2 mVs^−1^ (Fig. S7a) showed a lower current density compared to d-Ti_3_C_2_@NiCo_2_S_4_-24, likely due to the limited ion accessibility and reduced active surface area associated with the stacked multilayered MXene structure. Further, the CV profiles of d-Ti_3_C_2_@NiCo_2_S_4_-24 recorded at scan rates ranging from 2 to 25 mVs^−1^ (Fig. 4a) retain their shape and show a significant current response even at high scan rates, indicating excellent rate capability and capacitive behavior under fast charging conditions. This performance enhancement can be ascribed to the synergistic effects of the NiCo_2_S_4_ phase and the high conductivity of MXene, which together enable efficient charge transfer and robust structural stability. Collectively, these results establish d-Ti_3_C_2_@NiCo_2_S_4_-24 as the optimized electrode material with superior charge storage characteristics, which is further corroborated by galvanostatic charge–discharge (GCD) studies discussed in the following section.

The charge storage characteristics of the prepared electrodes were further evaluated through galvanostatic charge–discharge (GCD) measurements in 6 M KOH electrolyte within a potential window of 0–0.5 V versus Hg/HgO. The GCD curve of NiCo_2_S_4_-24 (Fig. S7c) recorded at a current density of 1 Ag^−1^ shows a discharge time of 435.5 s, corresponding to a specific capacity of 121.06 mAhg^−1^ (871 Fg^−1^), which is indicative of a battery-type behavior dominated by the redox activity of Ni and Co species. In contrast, the GCD curves of the composite electrodes d-Ti_3_C_2_@NiCo_2_S_4_-4, d-Ti_3_C_2_@NiCo_2_S_4_-16, d-Ti_3_C_2_@NiCo_2_S_4_-24 and d-Ti_3_C_2_@NiCo_2_S_4_-48 (Fig. 4b) reveal a significant enhancement in discharge duration and overall capacitance for the Ti_3_C_2_ MXene-integrated samples, with d-Ti_3_C_2_@NiCo_2_S_4_-24 demonstrating the highest discharge time of 685 s at 1 Ag^−1^, clearly outperforming both the bare and other composite counterparts. This superior performance is attributed to the optimized growth and integration of NiCo_2_S_4_ over the Ti_3_C_2_ MXene scaffold, combined with the hexagonal plate-like morphology featuring a well-defined layered architecture, which collectively facilitate rapid electron transport, enhanced ionic diffusion, and provide abundant electroactive sites for improved electrochemical performance. On the other hand, ML-Ti_3_C_2_@NiCo_2_S_4_-24 exhibited an inferior specific capacity of 103.14 mAhg^−1^ (742 Fg^−1^) at 1 Ag^−1^ (Fig. S7c) compared to its d-Ti_3_C_2_@NiCo_2_S_4_-24 counterpart. This reduced performance can be attributed to the restacked nature and limited surface accessibility of the multilayered Ti_3_C_2_ MXene, which hinder ion diffusion and restrict the exposure of active sites. In contrast, d-Ti_3_C_2_@NiCo_2_S_4_-24 demonstrated superior electrochemical behavior due to the expanded interlayer spacing and improved ion transport pathways offered by the delaminated few-layered MXene. Consequently, the d-Ti_3_C_2_ was selected in this study to enhance the overall charge storage capability. Furthermore at 1 Ag^−1^, NiCo_2_S_4_-24 delivered a specific capacity of 121.06 mAhg^−1^ (871 Fg^−1^), whereas d-Ti_3_C_2_@NiCo_2_S_4_-24 exhibited a substantially higher capacity of 161.94 mAhg^−1^ (1165 Fg^−1^). The GCD curves at higher current densities (2–10 A g^−1^) are shown in Fig. S8, demonstrating that d-Ti_3_C_2_@NiCo_2_S_4_-24 exhibits the longest discharge time even at 10 A g^−1^ compared to other time variant composites. Moreover, d-Ti_3_C_2_@NiCo_2_S_4_-24 exhibited superior rate capability, retaining ~ 81% of its capacity as the current density increased from 1 to 5 Ag^−1^ (Fig. 4c), which is significantly higher than d-Ti_3_C_2_@NiCo_2_S_4_-4 (~ 37%), d-Ti_3_C_2_@NiCo_2_S_4_-16 (~ 54%), d-Ti_3_C_2_@NiCo_2_S_4_-48 (~ 69%), and the NiCo_2_S_4_-24 (~ 15.22%), primarily due to its well-defined hexagonal plate-like layered morphology that promotes efficient ion diffusion and charge transfer kinetics. This enhanced electrochemical storage performance of the hexagonal plate like d-Ti_3_C_2_@NiCo_2_S_4_-24 composite can be attributed to its increased surface area and preferential exposure of the (311) facet. This observation is consistent with previous studies [58, 59], which have shown that the (311) plane in NiCo_2_S_4_ structures facilitates greater electrolyte accessibility and exposure of redox-active sites, thereby significantly improving overall energy storage performance. To probe the underlying electrochemical kinetics, electrochemical impedance spectroscopy (EIS) was carried out, and the Nyquist plots (Fig. 4d) fitted using an equivalent circuit model (Fig. S9) show that d-Ti_3_C_2_@NiCo_2_S_4_-24 possesses the lowest R_ct_ (3.5 Ω) and R_s_ (0.3 Ω), demonstrating faster ion/electron transport and minimal internal losses compared to other samples. Furthermore, long-term cycling stability test carried out at 5 Ag^−1^ for 20,000 cycles (Fig. 4e) shows that d-Ti_3_C_2_@NiCo_2_S_4_-24 retains 85.2% of its initial capacitance along with a coulombic efficiency of 99.8%, far superior to the 57% retention observed for NiCo_2_S_4_-24 (Fig. S7d). Post-cycling SEM analysis (inset of Fig. 4e) reveals that the d-Ti_3_C_2_@NiCo_2_S_4_-24 electrode retains its well-defined hexagonal morphology even after 20,000 charge–discharge cycles, closely matching the morphological characteristics observed in the pre-cycled SEM image (Fig. S10). In stark contrast, the pristine NiCo_2_S_4_ electrode undergoes pronounced structural deterioration within only 10,000 cycles, as evidenced by a clear comparison of the SEM images recorded before (Fig. S11a) and after (Fig. S11b) electrochemical cycling. These findings highlight the superior structural stability of the d-Ti_3_C_2_@NiCo_2_S_4_-24 architecture, which effectively mitigates electrode degradation under prolonged cycling conditions. Overall, the effect of using aqueous-dispersed Ti_3_C_2_ MXene in place of multilayered bulk Ti_3_C_2_ MXene, along with the influence of morphology control, has been systematically investigated. Results reveal that the dispersed Ti_3_C_2_ enhances specific capacitance by 57% compared to the bulk form. Furthermore, among the tested morphologies, the hexagonal structure demonstrates the most favorable performance, elevating the rate capability from 37% (in case of spherical morphology) and 69% (in case of mixed morphology) to 81%. A summary of the electrochemical performance of various NiCo_2_S_4_-based composite electrodes is provided in Table 1, showing that d-Ti_3_C_2_@NiCo_2_S_4_-24 exhibits better overall performance than most reported systems, with only a few achieving higher capacitance but lacking cycling stability.

**Table 1:** Comparison of electrochemical performances of different NiCo_2_S_4_-based electrodes

No	Sample name	Potential window	Capacitance	Long cycle retention (%)	Reference
1	d-Ti_3_C_2_@NiCo_2_S_4_-24	0.5 V	161.94 mAhg^−1^ (1165 Fg^−1^) at 1 Ag^−1^	85% after 20,000 cycles	This work
2	Nanostructured NiCo_2_S_4_	0.5 V	515.7 Fg^−1^ at 1 mA cm^−2^	99..9% after 2000 cycles	[61]
3	NiCo_2_S_4_@N-doped CNT	0.5 V	783.5 Fg^−1^ at 1 Ag^−1^	88.9% after 3000 cycles	[62]
4	NiCo_2_S_4_/Ni-rGO	0.45 V	879.01 Fg^−1^ at 0.5 Ag^−1^	91.6% after 10,000 cycles	[63]
5	PPy@NiCo_2_S_4_ nanosheets	0.4 V	8.5 Fcm^−2^ at 1 mAcm^−2^	84.4% after 1000 cycles	[64]
6	GNF/NCS/CNS	0.55 V	15.6 Fcm^−2^ at 10 mA/cm^2^	93% after 5000 cycles	[65]
7	NiCo_2_S_4_@nanoengineered NF	0.5 V	1223.8 Cg^−1^ at 2.5 Ag^−1^	94.56% after 10,000 cycles	[66]
8	NiCo_2_S_4_ nanosheets	0.45 V	1257.1 Fg^−1^ at 1 Ag^−1^	80% after 1000 cycles	[67]
9	NiCo_2_S_4_ nanowire	0.45 V	1454.6 Fg^−1^ at 1.3 Ag^−1^	96% after 3000 cycles	[68]
10	NiCo_2_S_4_/CNT	0.55 V	1765 Fg^−1^ at 1 Ag^−1^	71.7% after 5000 cycles	[69]
11	NiCo_2_S_4_/Ti_3_C_2_ MXene	0.5 V	2544.1 Fg^−1^ at 1 Ag^−1^	96% after 5000 cycles	[32]
12	Ti_3_C_2_ MXene/NiCo_2_S_4_	0.4 V	2675 Fg^−1^ at 1 Ag^−1^	96.51% after 10,000 cycles	[28]
13	CF-NCS	0.5 V	167.28 Fg^−1^ at 4 Ag^−1^	81% for 3000 cycles	[33]

*CNT Carbon nanotube, rGO Reduced graphene oxide, PPy Polypyrrole, GNF Graphene, NCS NiCo_2_S_4_ nanotube, CNS Co_x_Ni_(3−x)_S_2_ nanosheet, NF Nickel foam, CF Cu-foil

To validate the practical applicability of the optimized electrode, an asymmetric supercapacitor (ASC) device, denoted as d-Ti_3_C_2_@NiCo_2_S_4_-24 //AC, was assembled using d-Ti_3_C_2_@NiCo_2_S_4_-24 as the positive electrode and commercial activated carbon (AC) as the negative electrode in 6 M KOH aqueous electrolyte.

**Fig. 5:**
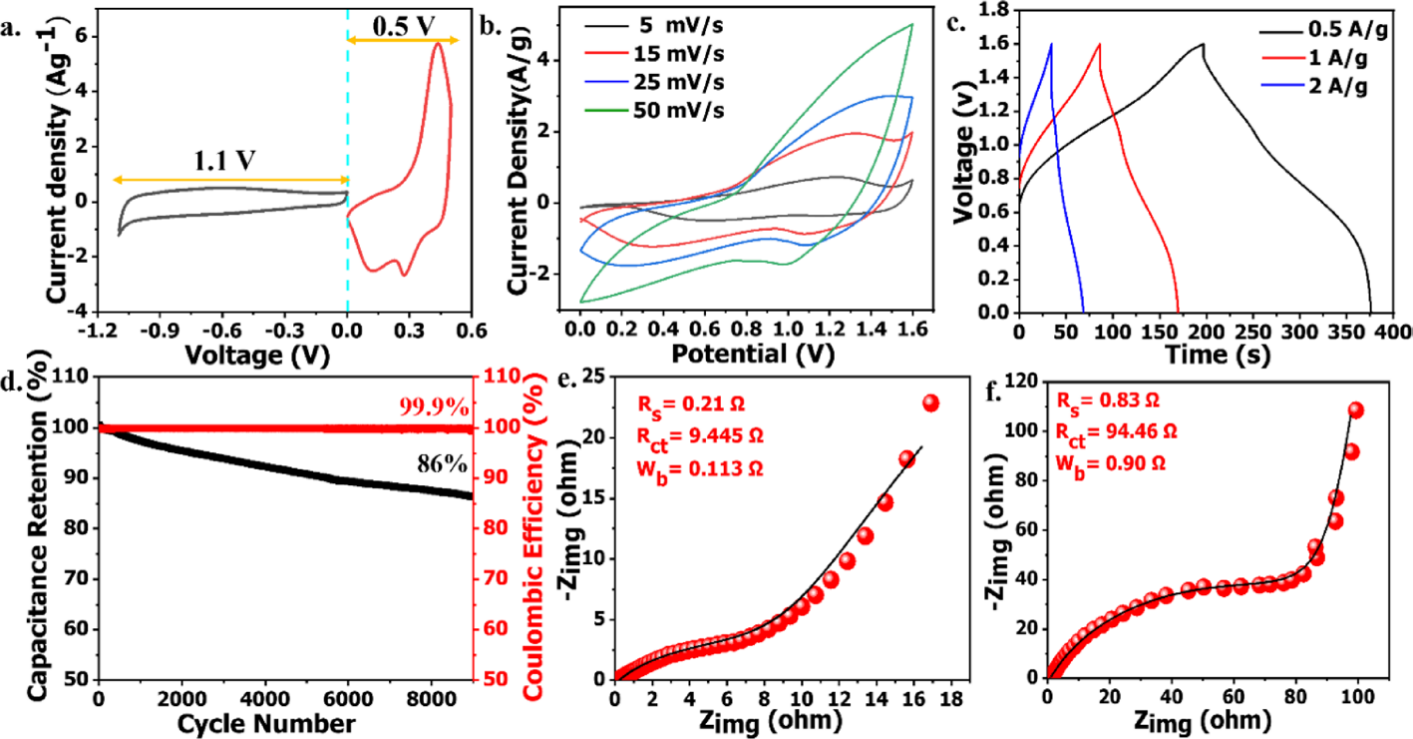
**a** Potential window determination CV curve d-Ti_3_C_2_@NiCo_2_S_4_-24//AC device, **b** CV curves of d-Ti_3_C_2_@NiCo_2_S_4_-24//AC device, **c** GCD curves of d-Ti_3_C_2_@NiCo_2_S_4_//AC device, **d** Cycle stability with Coulombic efficiency of d- Ti_3_C_2_@NiCo_2_S_4_-24//AC, **e** Before cycle nyquist plots of d-Ti_3_C_2_@NiCo_2_S_4_-24//AC device, **f** After cycle nyquist plots of d-Ti_3_C_2_@NiCo_2_S_4_-24//AC device

The CV and GCD curves of the AC electrode are presented in Fig. S12a, b, respectively, where CV was employed to determine the stable potential window of the negative electrode, while the capacitance values extracted from the GCD curves were utilized for mass balance calculations. The mass ratio between the positive and negative electrodes was optimized to 0.38:1 using the standard charge balance equation with detailed calculations provided in the Supporting Information. The optimal operating voltage window of the device was determined to be 1.6 V (d-Ti_3_C_2_@NiCo_2_S_4_-24: 0 to + 0.5 V; AC: 0 to − 1.1 V), as confirmed by the linear and non-distorted CV response shown in Fig. 5a. The CV curves at various scan rates (Fig. 5b) exhibit broad, well-defined redox peaks superimposed on a capacitive baseline, signifying the synergistic contribution of both pseudocapacitance (from d-Ti_3_C_2_@NiCo_2_S_4_-24) and EDLC (from AC) in the charge storage process. Even at elevated scan rates, the CVs retained their overall shape and area, highlighting excellent rate capability and charge transfer dynamics. GCD profiles recorded at current densities ranging from 0.5 to 2 Ag^−1^ (Fig. 5c) show quasi-symmetric charge–discharge curves with distinguishable voltage plateaus, indicating a dominant faradaic mechanism. The specific capacity were calculated to be 7.77 mAhg^−1^ (55.93 Fg^−1^), 7.3 mAhg^−1^ (52.5 Fg^−1^) at 1 Ag^−1^, and 5.91 mAhg^−1^ (42.5 Fg^−1^) at 2 Ag^−1^. Correspondingly, the device delivered a maximum energy density of 19.88 Whkg^−1^ at a power density of 399.82 Wkg^−1^. Long-term cycling stability was also evaluated, where the device retained 86% of its initial capacitance after 9000 cycles at 5 Ag^−1^, while maintaining a nearly ideal 99.9% coulombic efficiency, as shown in Fig. 5d. Electrochemical impedance spectroscopy (EIS) before and after cycling (Figs. 5e and 4f) revealed a slight increase in charge transfer resistance (R_ct_) from 9.445 to 94.46 Ω, a typical observation after extended operation, yet still indicative of stable interfacial properties. Though the present supercapacitor system is formed with a battery-type electrode materials however, any kind of significant pressure build-up and temperature change during the operation of the device is not observed which is essential for safe use of this type storage system. Figure S13 presents the Ragone plot, showing that d-Ti_3_C_2_@NiCo_2_S_4_-24//AC outperforms most similar material–based asymmetric supercapacitors by delivering higher energy density at comparable or higher power densities, highlighting its excellent electrochemical performance and practical applicability*.* Solid electrolyte systems could be explored to further enhance the energy density of d-Ti_3_C_2_@NiCo_2_S_4_-24//AC [70], however aqueous devices offer safer operation by avoiding significant temperature rise and preventing pressure buildup within the safe voltage window. These results affirm the promise of d-Ti_3_C_2_@NiCo_2_S_4_-24 as a high-performance battery-type material for next-generation aqueous ASC devices.

## Conclusion

In this work, a one-step hydrothermal strategy is adopted for the synthesis of d-Ti_3_C_2_@NiCo_2_S_4_ composites, where the influence of hydrothermal duration (4–48 h) on morphology and electrochemical performance is systematically investigated, revealing that precise control over morphology plays a pivotal role in enhancing capacitive behavior, an aspect not previously addressed in detail. The results confirmed that the morphology of d-Ti_3_C_2_@NiCo_2_S_4_ composites evolved from spherical agglomerates at 4 h, to partially developed hexagonal plates at 16 h, well-defined layered hexagonal plates at 24 h, and finally a mixture of irregular rod-like and deformed hexagonal structures at 48 h**.** Among the prepared composites, d-Ti_3_C_2_@NiCo_2_S_4_-24, exhibiting a well-defined hexagonal plate-like morphology with a layered architecture, delivered the highest specific capacity of 161.94 mAh g^−1^ (1165 F g^−1^) at 1 Ag^−1^, excellent rate capability (~ 81% retention at 5 Ag^−1^), and long-term cycling stability (85% retention over 20,000 cycles). The superior electrochemical performance was attributed to the synergistic effect of the delaminated Ti_3_C_2_ structure, offering enhanced ion accessibility and charge transport, and the controlled hexagonal morphology of the composite, which further facilitated efficient electrolyte penetration and abundant active sites. Comparative electrochemical evaluations confirmed the clear superiority of d-Ti_3_C_2_@NiCo_2_S_4_-24 over both NiCo_2_S_4_-24 and ML-Ti_3_C_2_@NiCo_2_S_4_-24 in terms of energy storage performance. Furthermore, the asymmetric device (d-Ti_3_C_2_@NiCo_2_S_4_-24//AC) achieved a wide operating voltage of 1.6 V, high energy and power densities, and retained 86% of its capacitance after 9000 cycles. Overall, this study highlights the critical role of MXene delamination and morphology engineering in designing high-performance, scalable electrode materials for next-generation energy storage applications.

## Supplementary Information

Below is the link to the electronic supplementary material.

Supplementary Material 1.

## Data Availability

Data supporting the findings of this study are available from the corresponding author upon reasonable request.
