# Supplementary material for: Ti3C2-Mxene-dispersion and morphology controlled battery-type nickel cobalt sulphide based nanocomposites for the application as aqueous asymmetric supercapacitor with improved rate
Source: Discov Nano. 2025 Nov 24;20(1):216. doi: 10.1186/s11671-025-04396-3 (PMC12644273; doi:10.1186/s11671-025-04396-3)
Supplement: Supplementary file 1 — Supplementary Material 1. [file 11671_2025_4396_MOESM1_ESM.docx]

**Supporting Information**

Ti_3_C_2_-Mxene-dispersion and Morphology Controlled Nickel Cobalt Sulphide Based Nanocomposites for the Application as Battery-type Aqueous Asymmetric Supercapacitor with Improved Rate

Abhinaba Das^1^***^†^***, Arnab Samanta Roy Choudhury^1^***^†^***and Pallab Bhattacharya^1,2^*

^1^ Functional Materials Group, Advanced Materials & Corrosion (AMC) Division, CSIR-National Metallurgical Laboratory (NML), Burmamines, East Singhbhum, Jamshedpur, Jharkhand-831007, India

^2^ Academy of Scientific and Innovative Research (AcSIR), Ghaziabad 201002,

India.

***^†^*** Abhinaba Das and Arnab Samanta Roy Choudhury contributed equally to

this paper.

*Corresponding author. Tel. /fax: 6295168707

*E-mail address: [pallab.nml@csir.res.in](mailto:pallab.nml@csir.res.in) (Pallab Bhattacharya)

Author’s email addresses: [abhinabad35@gmail.com](mailto:abhinabad35@gmail.com) (Abhinaba Das),

[arnabonii@gmail.com](mailto:arnabonii@gmail.com) (Arnab Samanta Roy Choudhury)

**1 Synthesis of Materials**

**1.1 Chemicals used**

A highly pure Ti_3_AlC_2_ MAX phase (~99%) was successfully synthesized in our laboratory on a large scale (>300 g) and used as the precursor for Ti_3_C_2_ MXene in this study. Nickel nitrate hexahydrate [Ni(NO_3_)_2_·6H_2_O], cobalt nitrate hexahydrate [Co(NO_3_)_2_·6H_2_O], and thiourea (CH_4_N_2_S) were employed as the sources of nickel, cobalt, and sulfur, respectively. Potassium hydroxide (KOH) was used for the preparation of the electrolyte. For electrode fabrication, polyvinylidene fluoride (PVDF) was utilized as a binder to enhance the adhesion of the active material, while carbon black was added to improve electrical conductivity. N-methyl-2-pyrrolidone (NMP, 99%) served as the solvent. Nickel foam (1 mm thick) was used as the current collector during electrochemical measurements. Commercial activated carbon (AC) was used as the negative electrode in the asymmetric device. All chemicals were purchased from Merck Chemicals, India, and used without further purification.

**2 Characterization and Instrumentation**

The phase and crystallographic structure of the synthesized samples were examined using a Bruker D8 DISCOVER semi-automatic X-ray diffractometer (XRD) with Cu Kα radiation (λ = 0.154178 nm), operating in the 2θ range of 6°–65° at a scan rate of 1° min^-1^. The instrument was operated at 50 kV accelerating voltage and an emission current of approximately 300 mA. The chemical composition was analyzed by X-ray photoelectron spectroscopy (XPS, SPECS, Germany) equipped with an Mg Kα twin anode X-ray source (E = 1253.6 eV). Binding energies were calibrated using the C 1s peak at 284.6 eV, and data analysis was conducted using CASA XPS software. Surface morphology and microstructural features were studied using scanning electron microscopy (SEM, Nova Nano SEM 430, Netherlands). High-resolution transmission electron microscopy (HR-TEM) was performed on a JEOL JEM-1230 at an accelerating voltage of 120 kV. Fourier-transform infrared (FT-IR) spectroscopy was carried out in the range of 400–1000 cm^-1^ using the KBr pellet technique to identify functional groups. Raman spectroscopy and mapping were performed using a WITec alpha300 R confocal Raman microscope equipped with a 532 nm laser source operated at 0.2 mW. Chat-GPT has been used for language editing to enhance the readability of the manuscript.

**3 Few layer & multilayer Ti_3_C_2_ ratio**

Total Ti_3_C_2_ taken (x) = 0.2g

After exfoliation (sonication & centrifuged) weight of sediment multilayered Ti_3_C_2_  remains is (y) g

The measured weight few layered Ti_3_C_2_ at dispersion (z) = 0.05 g

So, x = y + z

→ 0.2 g = y + 0.05 g

→ y = 0.2 g – 0.05 g

→ y = 0.15 g

The ratio of y : z = 0.15 : 0.05 = 3 : 1

**4 Electrode preparation:**

Electrochemical measurements were conducted using a three-electrode configuration on a Gamry Potentiostat (Model No: Reference 600+). The working electrodes were fabricated by mixing the active material, PVDF, and carbon black in an 8:1:1 weight ratio. A sufficient amount of NMP was added to form a uniform slurry, which was then coated onto nickel foam (1 cm² area), serving as the current collector. The coated electrodes were dried under vacuum at 60 °C for 14 hours. The average mass loading of active material was maintained at approximately ~7 mg cm^-2^. A graphite rod and a Hg/Hgo electrode were employed as the counter and reference electrodes, respectively. The electrochemical behavior of the electrodes was evaluated in 6 M KOH aqueous electrolyte using cyclic voltammetry (CV), galvanostatic charge–discharge (GCD), and electrochemical impedance spectroscopy (EIS).

**5 Equations used:**

The specific capacitance (C_s_), energy density (E_s_) and power density (P_s_) of the electrode were calculated from the obtained GCD data by using the following equations [1,2]

$C_{s}$= $\frac{I \times\Delta t}{m \times\Delta V}$ (1)

E_s_ = $\frac{C_{s} \times{\Delta V}^{2}}{2 \times3.6}$ (2)

P_s_ = $\frac{3600 \times E_{s}}{\Delta t}$ (3)

Where C_s_ (Fg^-1^) is the specific capacitance, I (A) is the current, ∆t (s) is the discharge time, m (g) is the mass loading of the respective electrode, potential window is denoted by ∆V (Vs^-1^) [3,4].

E_s_ (Whkg^-1^) and P_s_ (Wkg^-1^) are the gravimetric energy density and gravimetric power density of the electrode.

The aqueous asymmetric SC device was fabricated using d-Ti_3_C_2_@NiCo_2_S_4_-24 as the positive electrode and AC (activated carbon) as the negative electrode, denoted as d-Ti_3_C_2_@NiCo_2_S_4_-24//AC. To ensure optimized electrochemical performance of the d-Ti_3_C_2_@NiCo_2_S_4_-24//AC device, the mass of both electrodes was carefully adjusted based on the charge balance condition using the following equation [5]:

$\frac{m^{+}}{m^{-}}$ = $\frac{C^{-} \times\Delta V}{C^{+}\times\Delta V}$ (4)

Where m⁺ is the mass of the positive electrode (in g), m⁻ is the mass of the negative electrode (in g), C⁺ is the capacitance of the positive electrode (in F/g), C⁻ is the capacitance of the negative electrode (in F/g), and ΔV is the potential window (in V). Based on the charge balance calculation, the appropriate mass ratio of d-Ti_3_C_2_@NiCo_2_S_4_-24 (positive electrode) and AC (negative electrode) is 0.38:1 which was followed for fabricating the aqueous asymmetric SC having a separation distance of 2 cm between the two electrodes.

We examined the electrochemical performance of the asymmetric supercapacitor (SC), employing a 6M KOH solution as the electrolytic medium. To calculate the specific capacitance (C_s_) for each electrode, we employed the subsequent equation

$C_{s}$= $\frac{I \times\Delta t}{m \times\Delta V}$ (5)

Where I (A), Δt (s), m (g) and ΔV (V) represent the discharge current, discharge time, total mass of active material in both electrodes and the potential window of the negative and positive electrode, respectively. Furthermore, we determined the energy and power density of the device using the following equations

E_s_ = $\frac{C_{s} \times{\Delta V}^{2}}{2 \times3.6}$ (6)

P_s_ = $\frac{3600 \times E_{s}}{\Delta t}$ (7)

**6 Supplementary Figures**


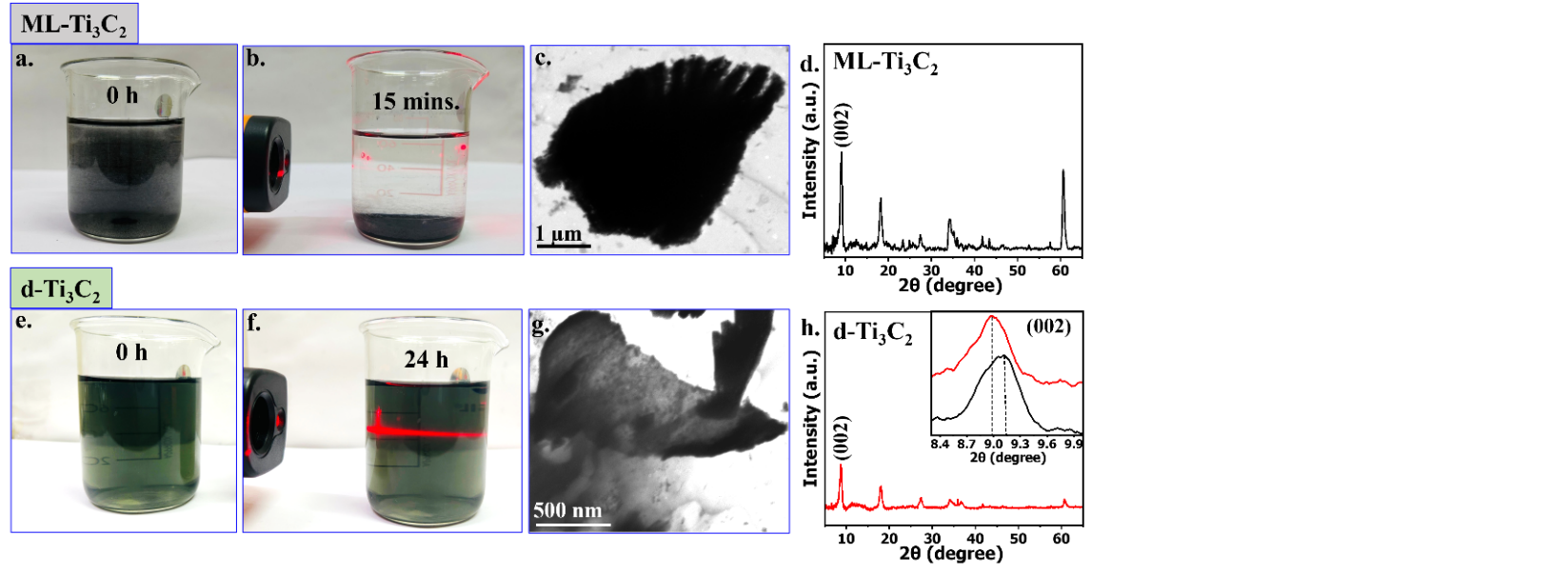


**Fig. S1 a** Solution of ML-Ti_3_C_2_ at 0 h, and **b** after 15 minutes **c** TEM image of ML-Ti_3_C_2_, **d** XRD of ML-Ti_3_C_2_, **e** Dispersed solution of d-Ti_3_C_2_ solution at 0 h, and **f** after 24h **g** TEM image of d-Ti_3_C_2_ **h** XRD of d-Ti_3_C_2_ (inset shows the magnified 002 peak of d-Ti_3_C_2_ and ML-Ti_3_C_2_ MXene).


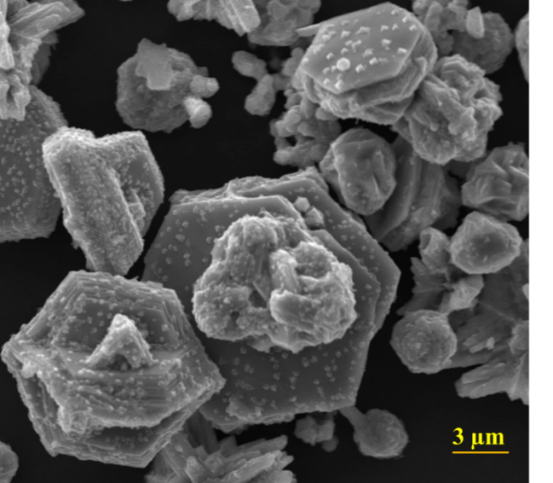


**Fig. S2** SEM image of d-Ti_3_C_2_@NiCo_2_S_4_-16.


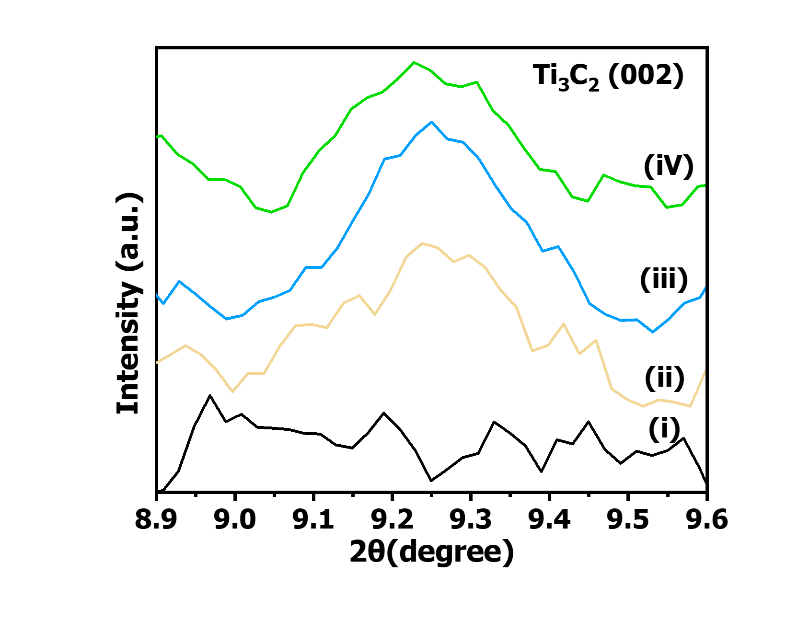


**Fig. S3** Magnified XRD of (i) NiCo_2_S_4_-24 (ii) d-Ti_3_C_2_@NiCo_2_S_4_-4, (iii) d-Ti_3_C_2_@NiCo_2_S_4_-16, (iv) d-Ti_3_C_2_@NiCo_2_S_4_-48.


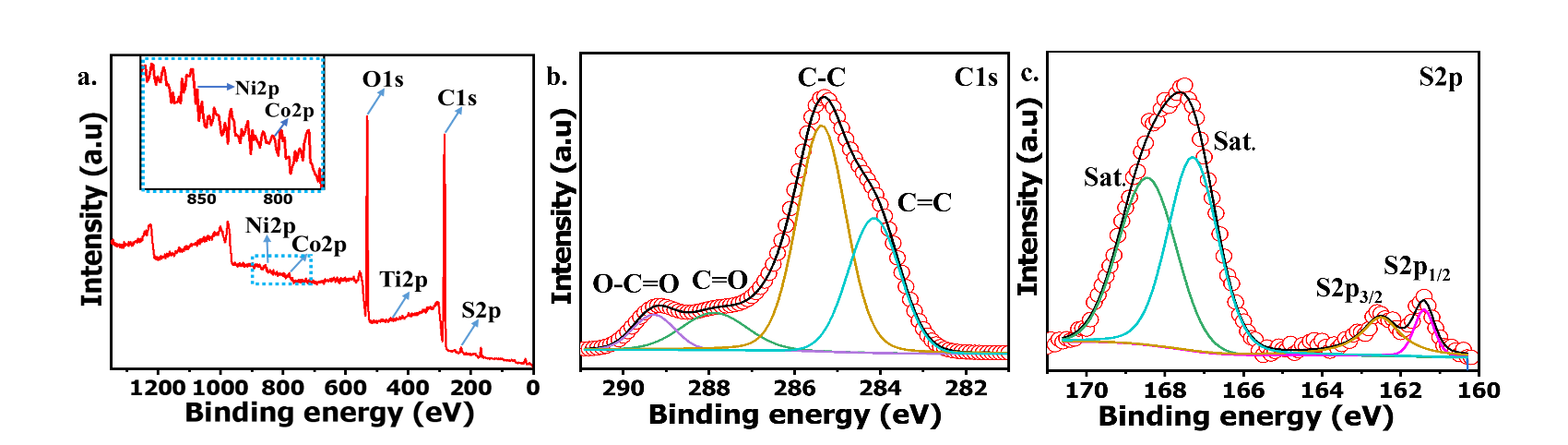


**Fig. S4** **a** Survey peak of d-Ti_3_C_2_@NiCo_2_S_4_-24, **b** XPS plot of C1s, **c** XPS plot of S2p.


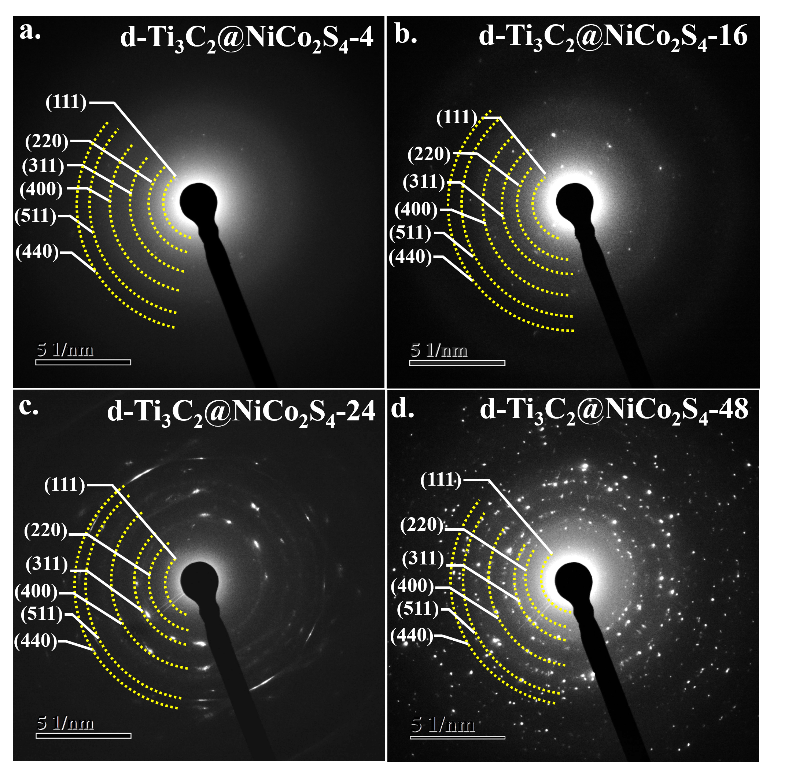


**Fig. S5 a** SAED image of d-Ti_3_C_2_@NiCo_2_S_4_-4, **b** d-Ti_3_C_2_@NiCo_2_S_4_-16, **c** d-Ti_3_C_2_@NiCo_2_S_4_-24, and **d** d-Ti_3_C_2_@NiCo_2_S_4_-48.


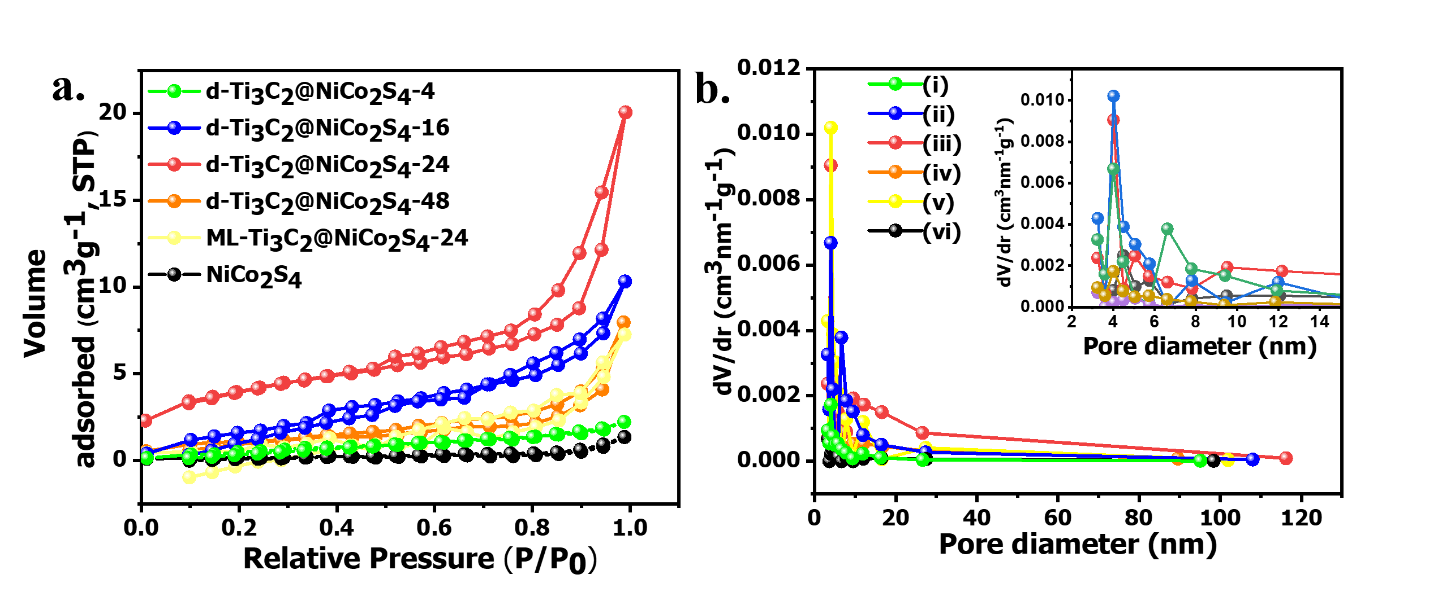


**Fig. S6 a** N_2_ adsorption−desorption isotherm, and **b** BJH pore size distribution plot of (i) d-Ti_3_C_2_@NiCo_2_S_4_-4, (ii) d-Ti_3_C_2_@NiCo_2_S_4_-16, (iii) d-Ti_3_C_2_@NiCo_2_S_4_-24, (iv) d-Ti_3_C_2_@NiCo_2_S_4_-48, (v) ML-Ti_3_C_2_@NiCo_2_S_4_-24, (vi) NiCo_2_S_4_-24


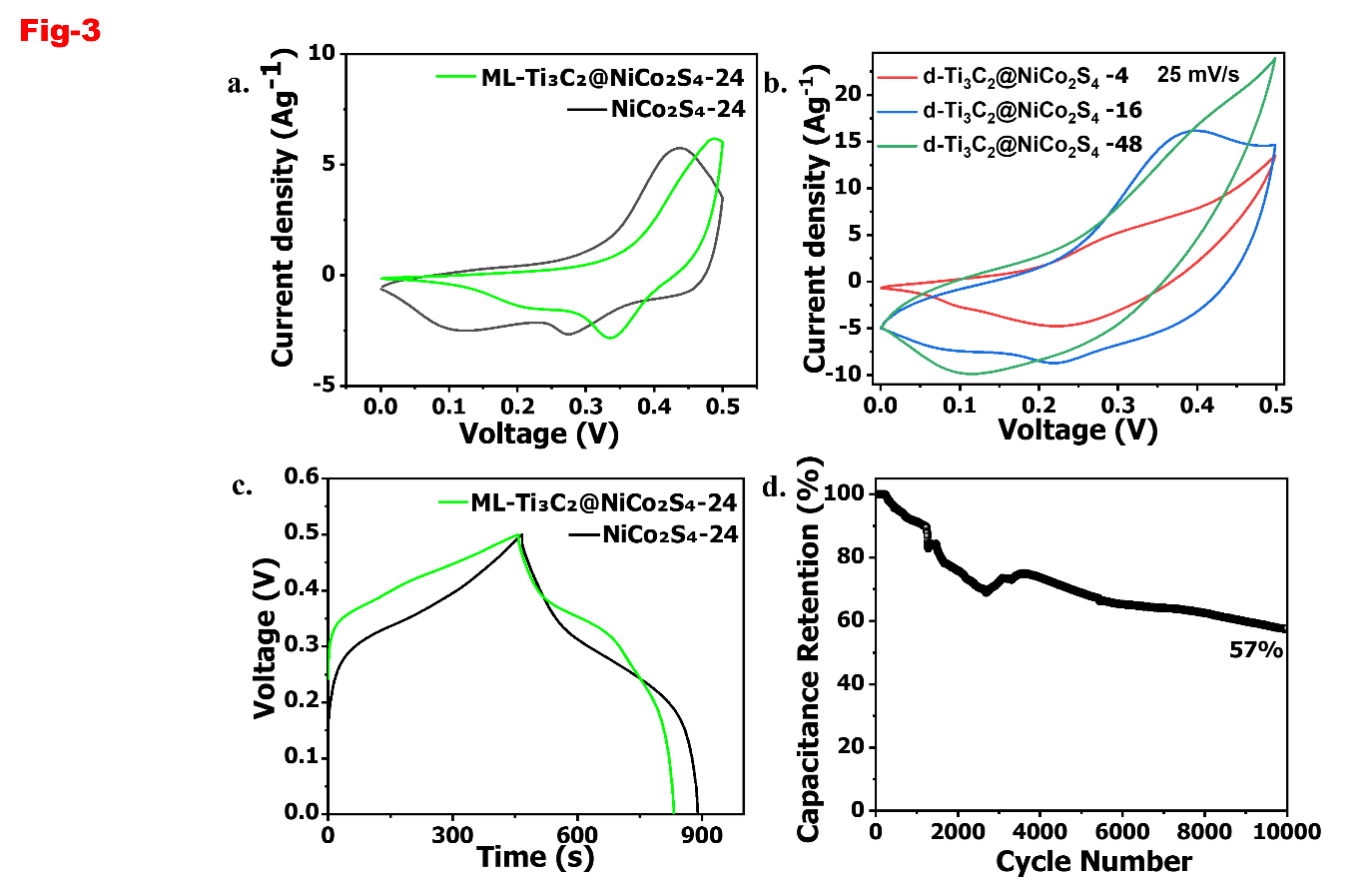


**Fig. S7 a** CV of ML-Ti_3_C_2_@NiCo_2_S_4_-24 & NiCo_2_S_4_-24 at 2 mVs^-1^, **b** CV of d-Ti_3_C_2_@NiCo_2_S_4_-4, d-Ti_3_C_2_@NiCo_2_S_4_-16, d-Ti_3_C_2_@NiCo_2_S_4_-48 at 25 mVs^-1^, **c** GCD of ML-Ti_3_C_2_@NiCo_2_S_4_-24 & NiCo_2_S_4_-24 at 1 Ag^-1^, **d** Cycle stability of NiCo_2_S_4_-24


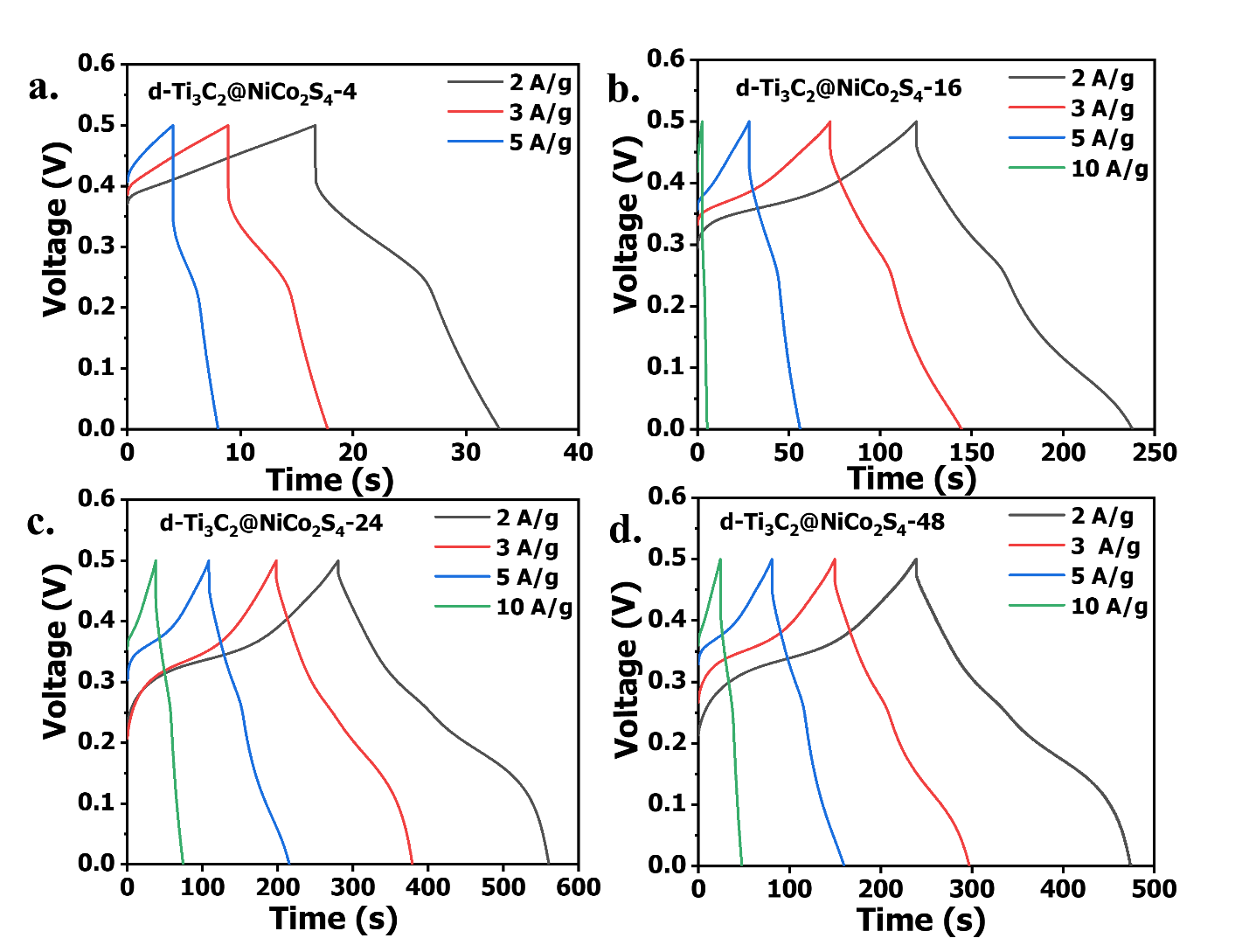


**Fig. S8 a** GCD curves of d-Ti_3_C_2_@NiCo_2_S_4_-4 electrode at current densities of 2, 3, and 5 A g⁻¹; **b** GCD curves of d-Ti_3_C_2_@NiCo_2_S_4_-16 electrode at current densities of 2, 3, 5 and 10 A g⁻¹; **c** GCD curves of d- d-Ti_3_C_2_@NiCo_2_S_4_-24 electrode at current densities of 2, 3, 5 and 10 A g⁻¹; **d** GCD curves of d- d-Ti_3_C_2_@NiCo_2_S_4_-48 electrode at current densities of 2, 3, 5 and 10 A g⁻¹.


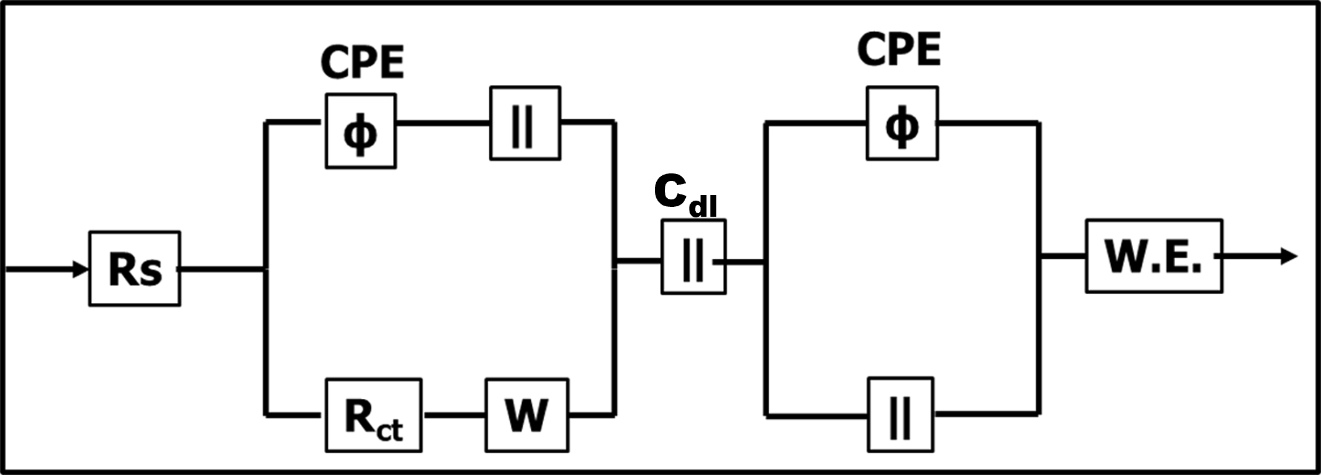


**Fig. S9** EIS circuit model used in Nyquist plot fitting.


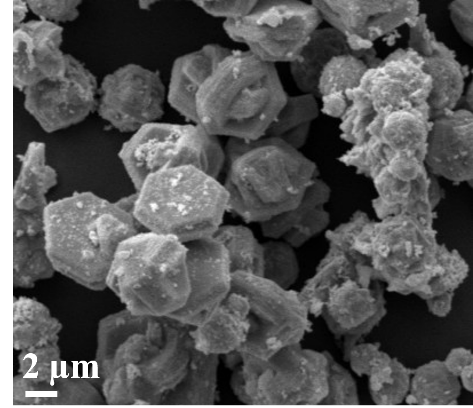


**Fig. S10** Pre-cycle SEM image of the d-Ti_3_C_2_@NiCo₂S₄-24 electrode


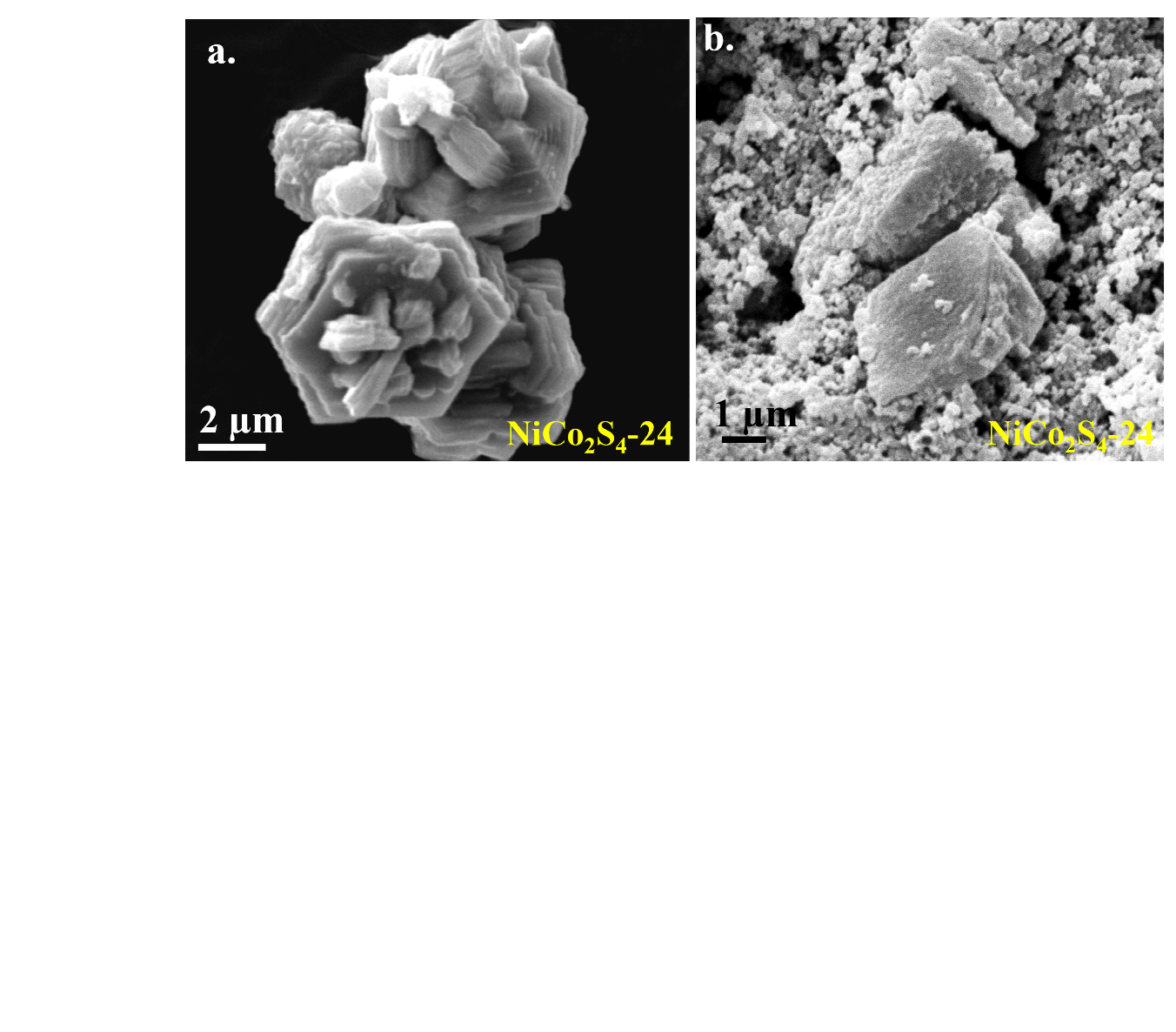


**Fig. S11** **a** SEM image of NiCo_2_S_4_-24 initially **b** SEM image of NiCo_2_S_4_-24 electrode after 10,000 cycles


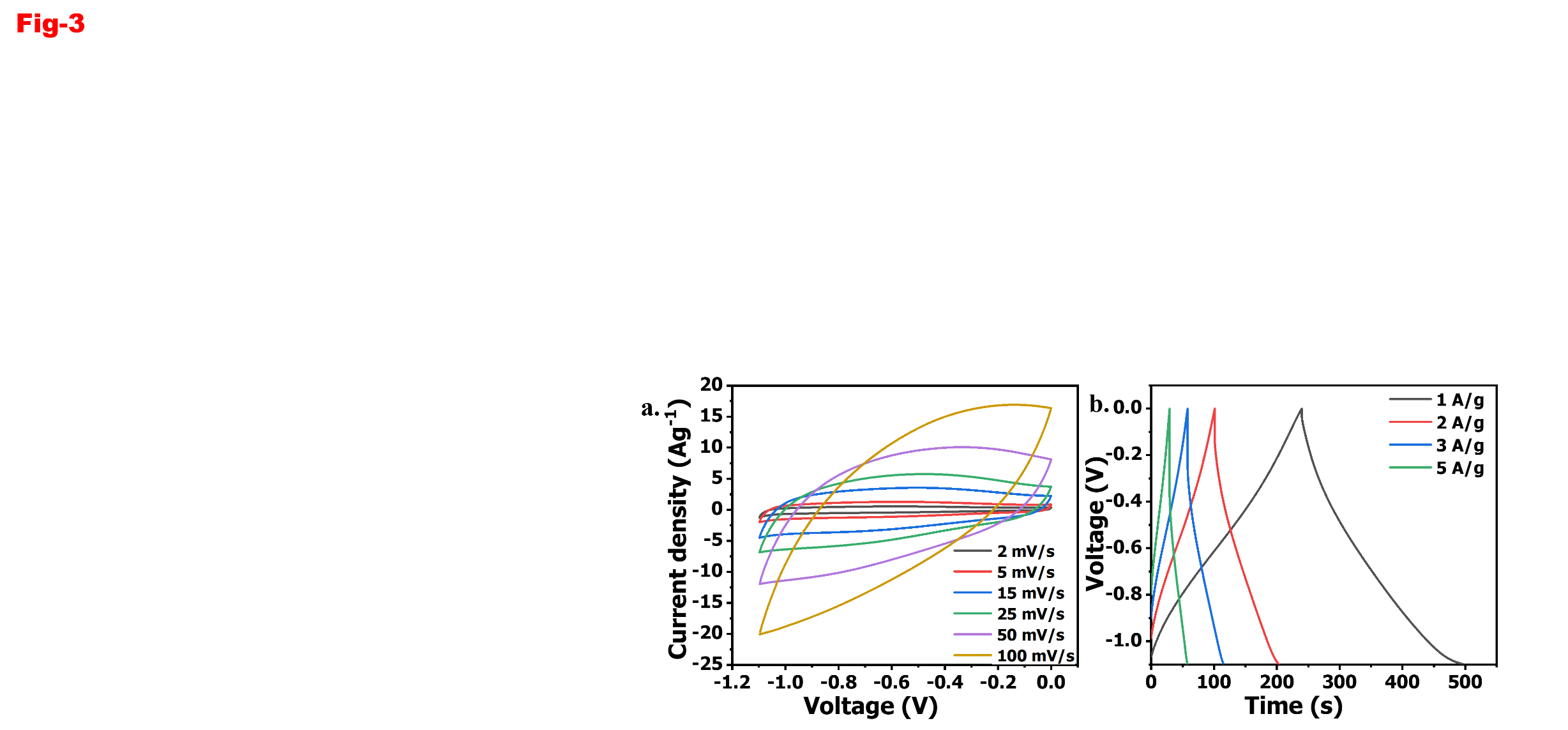


**Fig. S12 a** CV of Activated carbon (AC) in various scan rate, and **b** GCD of Activated carbon (AC) in various current densities


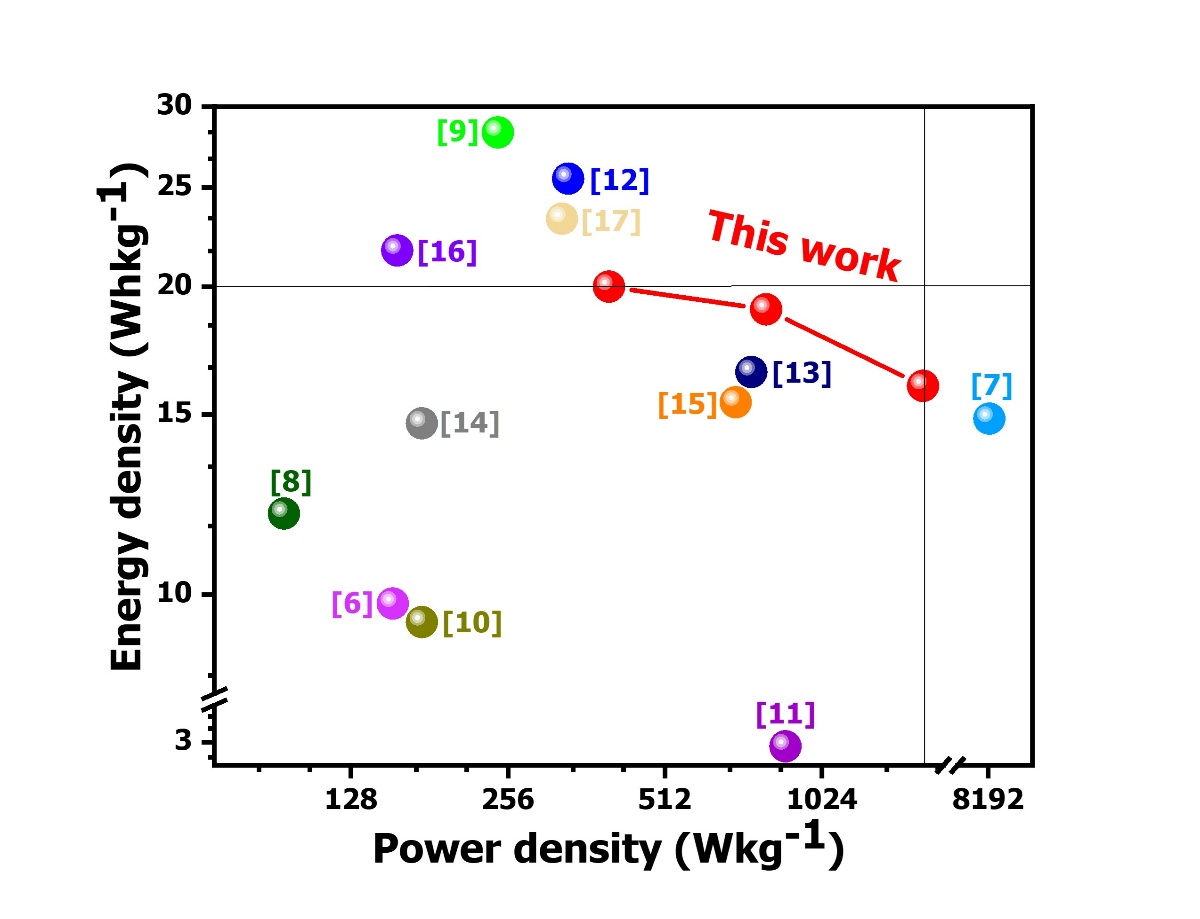


**Fig. S13** Ragone plot comparing the energy and power densities of the d-Ti_3_C_2_@NiCo_2_S_4_//AC device with related devices

**References**

[1] Ratha S, Sahoo S, Mane P, Polai B, Sathpathy B, Chakraborty B, et al. Experimental and computational investigation on the charge storage performance of a novel Al_2_O_3_-reduced graphene oxide hybrid electrode. Sci Rep. 2023;13. <https://doi.org/10.1038/s41598-022-23574-2>.

[2] Hameed BH, Tan IAW, Ahmad AL. Preparation of oil palm empty fruit bunch-based activated carbon for removal of 2,4,6-trichlorophenol: Optimization using response surface methodology. J Hazard Mater. 2008;164:1316–24. <https://doi.org/10.1016/j.jhazmat.2008.09.042>.

[3] Alam MdZ, Ameem ES, Muyibi SA, Kabbashi NA. The factors affecting the performance of activated carbon prepared from oil palm empty fruit bunches for adsorption of phenol. Chem Eng J. 2009;155:191–8. <https://doi.org/10.1016/j.cej.2009.07.033>.

[4] Yakout SM, El-Deen GS. Characterization of activated carbon prepared by phosphoric acid activation of olive stones. Arabian J Chem. 2011;9:S1155–62. <https://doi.org/10.1016/j.arabjc.2011.12.002>.

[5] Bi X, Li M, Zhou G, Liu C, Huang R, Shi Y, et al. High-performance flexible all-solid-state asymmetric supercapacitors based on binder-free MXene/cellulose nanofiber anode and carbon cloth/polyaniline cathode. Nano Res. 2023;16:7696–709. https://doi.org/10.1007/s12274-023-5586-1.

[6] Senthilkumar ST, Selvan RK. Fabrication and performance studies of a cable-type flexible asymmetric supercapacitor. Phys Chem Chem Phys. 2014;16:15692. <https://doi.org/10.1039/c4cp00955j>.

[7] Pathak M, Polaki SR, Rout CS. High performance asymmetric supercapacitors based on Ti_3_C_2_T_x_ MXene and electrodeposited spinel NiCo_2_S_4_ nanostructures. RSC Adv. 2022;12:10788–10799. <https://doi.org/10.1039/d2ra00991a>.

[8] Tang C, Tang Z, Gong H. Hierarchically porous Ni-Co oxide for high reversibility asymmetric Full-Cell supercapacitors. J Electrochem Soc. 2012;159:A651–A656. <https://doi.org/10.1149/2.074205jes>.

[9] Zhu Y, Wu Z, Jing M, Yang X, Song W, Ji X. Mesoporous NiCo_2_S_4_ nanoparticles as high-performance electrode materials for supercapacitors. J Power Sources. 2014;273:584–590. <https://doi.org/10.1016/j.jpowsour.2014.09.144>.

[10] Kuang M, Wen ZQ, Guo XL, Zhang SM, Zhang YX. Engineering firecracker-like beta-manganese dioxides@spinel nickel cobaltates nanostructures for high-performance supercapacitors. J Power Sources. 2014;270:426–433. <https://doi.org/10.1016/j.jpowsour.2014.07.144>.

[11] Gao Y, Lin Q, Zhong G, Fu Y, Ma X. Novel NiCo_2_S_4_/graphene composites synthesized via a one-step in-situ hydrothermal route for energy storage. J Alloys Compd. 2017;704:70–78. <https://doi.org/10.1016/j.jallcom.2017.01.304>.

[12] Wu Z, Pu X, Ji X, Zhu Y, Jing M, Chen Q, Jiao F. High energy density asymmetric supercapacitors from mesoporous NICO2S4 nanosheets. Electrochim Acta. 2015;174:238–245. <https://doi.org/10.1016/j.electacta.2015.06.011>.

[13] Dang T, Wang L, Wei D, Zhang G, Li Q, Zhang X, Cao Z, Zhang G, Duan H. Bifunctional phosphorization synthesis of mesoporous networked Ni-Co-P/phosphorus doped carbon for ultra-stable asymmetric supercapacitors. Electrochim Acta. 2019;299:346–356. <https://doi.org/10.1016/j.electacta.2018.12.176>.

[14] Ding R, Qi L, Jia M, Wang H. Facile and large-scale chemical synthesis of highly porous secondary submicron/micron-sized NiCo2O4 materials for high-performance aqueous hybrid AC-NiCo_2_O_4_ electrochemical capacitors. Electrochim Acta. 2013;107:494–502. <https://doi.org/10.1016/j.electacta.2013.05.114>.

[15] Lu X-F, Wu D-J, Li R-Z, Li Q, Ye S-H, Tong Y-X, Li G-R. Hierarchical NiCo_2_O_4_ nanosheets@hollow microrod arrays for high-performance asymmetric supercapacitors. J Mater Chem A. 2014;2:4706–4713. <https://doi.org/10.1039/c3ta14930g>.

[16] Cheng D, Yang Y, Xie J, Fang C, Zhang G, Xiong J. Hierarchical NiCo_2_O_4_@NiMoO_4_ core–shell hybrid nanowire/nanosheet arrays for high-performance pseudocapacitors. J Mater Chem A. 2015;3:14348–14357 <https://doi.org/10.1039/c5ta03455h>.

[17] Wang X, Liu WS, Lu X, Lee PS. Dodecyl sulfate-induced fast faradic process in nickel cobalt oxide–reduced graphite oxide composite material and its application for asymmetric supercapacitor device. J Mater Chem. 2012;22:23114. <https://doi.org/10.1039/c2jm35307e>.
